# Unveiling Hepatic Protein Alterations in Neonatal and Infant Biliary Atresia

**DOI:** 10.1002/cpt.70244

**Published:** 2026-03-04

**Authors:** Zubida M. Al‐Majdoub, Martyn Howard, Brahim Achour, Jill Barber, Naved Alizai, Amin Rostami‐Hodjegan

**Affiliations:** ^1^ Centre for Applied Pharmacokinetic Research (CAPKR), University of Manchester Manchester UK; ^2^ Department of Biomedical and Pharmaceutical Sciences College of Pharmacy, the University of Rhode Island Kingston Rhode Island USA; ^3^ Leeds Teaching Hospitals NHS Trust Leeds UK; ^4^ Certara Predictive Technologies, Certara Inc Sheffield UK

## Abstract

Pediatric populations differ from adults in drug elimination capacity. While current scaling methods account for enzyme and transporter maturation, they overlook comorbidities, such as biliary atresia (BA), a liver disease appearing within the first 2–8 weeks of life that can progress to cirrhosis. Such conditions may impair hepatic drug clearance, requiring dose adjustments. Physiologically based pharmacokinetic (PBPK) tools aim to address such cases and have been advocated to fill gaps in clinical data instead of less formalized and evidence‐based guesswork. However, the paucity of systems data in rare disease populations has hindered the development of robust PBPK models. This study used global liquid chromatography and tandem mass spectrometry (LC–MS/MS) proteomics to quantify drug‐metabolizing enzymes and transporters in diseased neonatal (*n* = 13) and infant (*n* = 12) liver samples, revealing significant expression changes in biliary atresia (BA) livers vs. controls (*n* = 19). Based on cohort means, CYP2A6, CYP2B6, and CYP2E1 levels were 6–17‐fold higher in BA livers compared to controls, while CYP4F11 and CYP20A1 were reduced. UGT1A1, UGT2B4, and UGT2B7 showed up to 16‐fold higher abundance in neonates with BA. Among transporters, ABCF1 abundance increased dramatically (46‐fold), whereas B3AT/SLC4A1, ADT1/SLC25A4, and S27A5/SLC27A5 were decreased. The observed alterations suggest that assuming similar liver function in BA and non‐BA patients has implications, with impact varying by drug clearance pathway. While in silico models can explore this, clinical pharmacokinetic studies in BA are essential for verification. To our knowledge, such studies are absent. Our observations underscore the urgent need for dedicated pharmacokinetic studies in BA patients to improve precision dosing.

**Study Highlights**

**WHAT IS THE CURRENT KNOWLEDGE ON THE TOPIC?**

Physiologically based pharmacokinetic (PBPK) modeling is increasingly used to support pediatric drug dosing, but its application in rare pediatric liver diseases like biliary atresia (BA) is limited by a lack of systems data. While the ontogeny of drug‐metabolizing enzymes and transporters is being characterized in healthy pediatric populations, little is known about how liver disease alters protein expression in neonates and infants.

**WHAT QUESTION DID THIS STUDY ADDRESS?**

This study investigated how the abundance of key hepatic drug‐metabolizing enzymes and transporters differs in neonatal and infant livers with BA compared to age‐matched controls, using quantitative global proteomics.

**WHAT DOES THIS STUDY ADD TO OUR KNOWLEDGE?**

This is the most comprehensive proteomic analysis to date in BA livers, revealing non‐uniform but significant alterations in protein expression. Several enzymes (e.g., CYP2A6, UGT2B7) and transporters (e.g., ABCF1) were markedly increased, while others (e.g., CYP4F11, SLC4A1) were decreased. These findings indicate that liver function in BA is selectively altered rather than globally diminished.

**HOW MIGHT THIS CHANGE CLINICAL PHARMACOLOGY OR TRANSLATIONAL SCIENCE?**

The data provide essential systems input for PBPK model development in neonates and infants with BA. This could improve model‐informed dosing strategies, helping to optimize drug exposure, and minimize toxicity in this vulnerable patient population.

The liver plays a critical role in drug metabolism, bioavailability and biliary excretion. Variations in the expression and activity of hepatic enzymes and transporters, along with factors such as hepatic blood flow, plasma protein binding, and biliary excretion, can significantly alter the pharmacokinetics,^1^, ^2^ particularly in patients with liver disease. Studies consistently show that inability to effectively metabolize drugs is impacted by the type and severity of liver disease.^1^, ^2^, ^3^ For instance, studies of adult patients with cirrhosis have revealed that 20% of drugs were inappropriately prescribed relative to liver function, and 30% led to adverse drug reactions, of which 80% were considered preventable.^2^, ^3^, ^4^ Some conditions, including pregnancy, demonstrate increased expression of some enzymes, such as CYP3A, leading to higher dose requirements to achieve similar systemic exposure. These findings emphasize the need for individualized dosing strategies that account for disease‐specific effects on clearance pathways.^5^ Pediatric drug dosing regimens are typically extrapolated from the “average” adult Caucasian male patient data and are rarely included in product labelling or the summary of product characteristics prior to market approval. This gap is particularly pronounced in younger pediatric populations with liver disease.^6^ While the British National Formulary (BNF) offer supplementary dosing guidance, adjustments are largely dependent on the clinician's expertise and experience rather than informed from data generated prospectively from a pediatric cohort. This underscores the need for robust pediatric‐specific pharmacokinetic studies and evidence‐based dosing frameworks to optimize drug safety and efficacy in these vulnerable populations, who are more susceptible to drug–drug interactions,^7^ adverse drug reactions or ineffective treatment.^8^, ^9^ Pharmacokinetic modeling and simulation tools are increasingly being used^10^ to reduce gaps in knowledge related to precision dosing for pediatric populations in the absence of clinical data. However, the availability of systems data that are needed to develop robust models to account for potential changes in many of these patient populations remains a key hurdle. Data on the ontogeny of drug‐metabolizing enzymes and transporters, as well as the impact of liver disease in pediatric populations, remain scarce. Although several studies have made progress in addressing ontogeny profiles of these proteins, their findings are often constrained by limited availability of age appropriate human samples and inconsistencies in study methodologies across laboratories.^11^, ^12^, ^13^, ^14^, ^15^ Biliary atresia (BA) is a rare liver disease, affecting approximately 1 in 5000 to 20,000 live births worldwide. It is characterized by inflammatory obstruction of the bile ducts, resulting in bile accumulation and If untreated, leads to rapid progression to cirrhosis and end stage liver disease.^16^, ^17^, ^18^ Symptoms such as jaundice, pale stools, and dark urine typically manifest soon after birth, with diagnosis confirmed through ultrasound, liver biopsy, and cholangiogram, depending on local protocols.^19^, ^20^, ^21^ The Kasai portoenterostomy, introduced in 1957, remains the primary surgical treatment to restore bile flow by connecting the liver directly to the intestine.^22^ This procedure delays the need for liver transplantation in many patients.^23^ Although BA is often associated with impaired liver function, recent transcriptomic and proteomic data,^24^, ^25^, ^26^ including our own proteomic data, indicate that this does not universally translate to reduced hepatic metabolic capacity. In fact, BA livers show marked increases in the expression of several drug‐metabolizing enzymes and transporters, suggesting that the functional capacity of the liver may be selectively altered rather than globally diminished. These findings challenge the assumption of uniformly reduced hepatic metabolic capacity in BA and underscore the need for disease‐specific evaluation to optimize drug dosing in this vulnerable pediatric population. Although no specific medication exists to directly manage biliary atresia, various drugs are commonly used pre‐ and post‐operatively to help biliary production and drainage, including prophylactic antibiotics^17^, ^18^, ^27^ and analgesics, such as morphine and paracetamol for pain management.^28^ As previously highlighted, drug dosing guidance for this orphan disease population is limited, necessitating careful monitoring when prescribing medications, particularly in infants under 2 months of age and those with hepatic impairment.^6^ The aim of this study was to quantify the abundance of key drug‐metabolizing enzymes and transporters in liver tissue from neonates and infants with BA. Leveraging a unique opportunity to compare these samples against age‐matched controls (non‐liver disease tissue), we utilized a label‐free quantitative proteomic analysis using the total protein approach (TPA)^29^ that also enabled global proteomic profiling. To the best of our knowledge, this study represents the most comprehensive analysis of proteomic changes in cytochrome P450 enzymes (CYPs), UDP‐glucuronosyltransferases (UGTs), and drug transporters in patients with BA to date.

## MATERIALS AND METHODS

All materials and chemicals were supplied by Sigma‐Aldrich (Poole, Dorset, UK), unless otherwise indicated.

### Human liver samples and tissue fractionation

Liver tissue from biliary atresia patients (*n* = 25; neonate = 13, infant = 12) obtained during Kasai portoenterostomy from Ethical Tissue University of Bradford Biobank (Approval Reference 18/LO/1969) and control, non‐liver disease associated tissue samples (*n* = 19, neonate = 13, infant = 6) from Erasmus University Medical Centre. Anonymized demographic data for the donors where available can be found in **Tables** **S1** **and** **S2**. A detailed description of the experimental procedures, including liver tissue processing and microsomal preparation, is provided in **Supporting Methods**.

### Sample preparation for proteomics

Solubilization and reduction of each microsomal fraction was achieved using sodium deoxycholate (final concentration 10% w/v) and DTT (final concentration 0.1 M), incubated at room temperature for 10 min, followed by 30 min at 56°C. Protein digestion was carried out using the filter‐aided sample preparation (FASP) method, as described in our previous publications.^30^, ^31^ Detailed protocols for protein digestion using the FASP method, and peptide clean‐up prior to liquid chromatography and tandem mass spectrometry (LC–MS/MS) analysis are provided in **Supporting Methods**.

### Liquid chromatography and tandem mass spectrometry (LC–MS/MS)

Peptide samples were analyzed by LC–MS/MS using an Ultimate® 3000 RSLC system coupled to a Q Exactive HF mass spectrometer. See **Supporting Methods** for additional details.

### LC–MS/MS data analysis

Data analysis was carried out in Progenesis v4.0 (Nonlinear Dynamics, Newcastle‐upon‐Tyne, UK) and the Mascot search engine. Full details of database searching parameters and data processing are provided in the **Supporting Methods**.

### Protein identification and quantification

The results obtained from Mascot were analyzed using a customized database based on the Uniprot Human Protein fasta file (https://www.uniprot.org/proteomes/UP000005640). Polymorphic proteins (e.g., CYP2C9) were reported only when variant‐specific peptides were confidently detected by LC–MS/MS; otherwise, proteins are presented as non‐polymorphic forms. Full details are provided in the **Supporting Methods**.

### Statistical analysis

All statistical analysis of the data was performed using Microsoft Excel v16.23 and GraphPad Prism v9.2.1 (La Jolla, California, USA). Mann–Whitney *U* tests were used to examine differences between diseased and control groups, and among age groups. Changes were deemed statistically significant if the *P*‐value was below 0.05.

## RESULTS

### General outcomes

The data were examined to quantify the CYPs, UGTs, and transporter proteins relevant to drug metabolism and disposition. Of these 24 CYPs, 9 UGTs, 10 ABC transporters, and 9 SLC transporters were quantified using the total protein approach (TPA) across the biliary atresia and control samples for both neonate and infant populations. Overall expression of these proteins in the majority of biliary atresia samples was high compared with controls.

### Expression of CYP enzymes in control and BA livers from neonates

**Figure 1:**
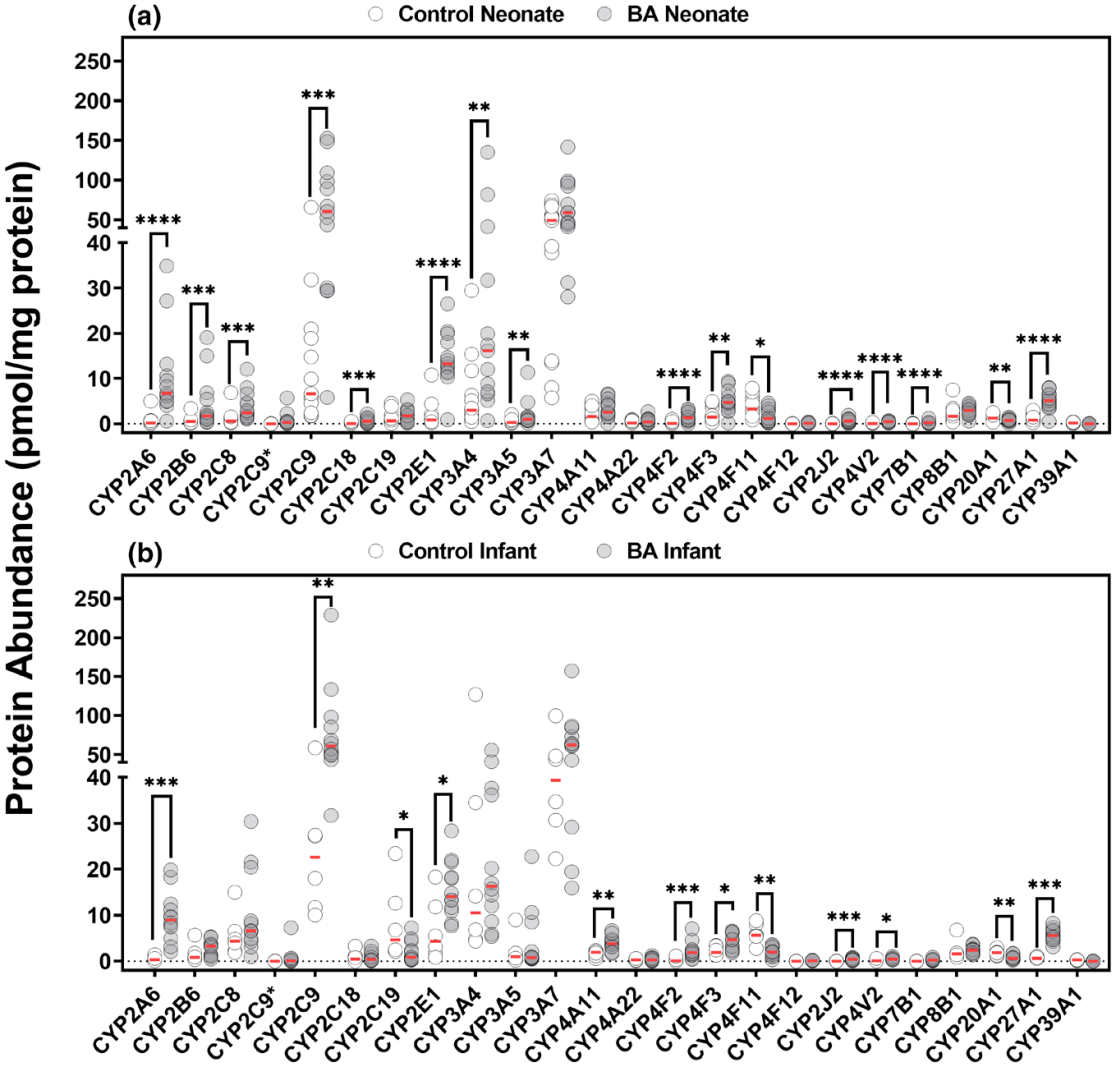
Individual abundance values of CYP enzymes in pmol/mg protein from control compared with biliary atresia (BA) neonatal (a) and infant livers (b). Expression levels in neonatal livers, including 13 control livers (open circles) and 13 BA livers (closed circles). Expression levels in infant livers, including 6 control livers (open circles) and 12 BA livers (closed circles). Statistical significance between control and BA groups is indicated by asterisks (*P < 0.05, **P < 0.01, ***P < 0.001, **P < 0.0001). The horizontal red lines represent median values. Protein abundance is expressed in pmol/mg protein.

The protein expression data revealed significant differences in both magnitude and statistical significance between control and BA neonate groups across several enzymes. Overall, mean expression levels were notably higher in the BA group compared with controls, with CYP2C9, CYP3A4, and CYP2E1 showing particularly large mean differences of 56, 23, and 12 pmol/mg protein, respectively (**Figure** **1** **a**). CYP2A6, CYP2E1, CYP2J2, CYP4F2, CYP4V2, CYP7B1, and CYP27A1 differed more strongly between groups (*P* < 0.0001). Additional enzymes, including CYP2B6, CYP2C8, CYP2C9* (rs3758581), CYP2C18, and CYP20A1, also differed between groups, with p‐values ranging from 0.0002 to 0.002. In contrast, CYP2C19, CYP3A7, CYP4A11, CYP4A22, and CYP8B1 showed no clear difference (*P* > 0.05). In terms of fold change, CYP2A6 (> 17 fold), CYP2B6, CYP2E1 (> 6 fold), and CYP4F2 (>8 fold) displayed higher expression in the BA group. Meanwhile, CYP2C8, CYP2C9, CYP2C18, CYP3A4, and CYP3A5 showed slightly lower changes in BA group (< 5‐fold increase) (**Figure** **1** **a**, **Table** **1**). The CV was generally lower in BA group for few enzymes, such as CYP2C9 (66%), CYP2E1 (43%), and CYP4F2 (68%), indicating more consistent expression levels (**Table** **1**). However, some enzymes, were expressed at negligible levels in the control group, such as CYP2C18 (0.12 pmol/mg protein), or no expression (CYP2C9* and CYP4F12) in the control group, limiting direct comparisons. Other proteins, such as CYP2B6, CYP3A4, and CYP3A7, showed more consistent CV% values between the two groups, suggesting less pronounced variability changes (**Figure** **1** **a**, **Table** **1**). Interestingly, CYP4F11, CYP20A1, and CYP39A1 had higher expression in controls, with BA livers showing decreased or absent expression. Mean expression values for these three enzymes in control livers were 3.88, 1.46, and 0.17 pmol/mg protein, respectively, while in BA livers, the mean dropped to 1.74, 0.75, and below the limit of quantification, respectively. The reversed trend compared with other enzymes highlights that BA may suppress some enzymes, such as CYP4F11, while upregulating or maintaining the expression of other CYP4F enzymes.

**Table 1:** Expression levels of 24 CYP enzymes in neonatal and infant livers under control and biliary atresia (BA) conditions

Enzyme	Donors	Median (pmol/mg)	Mean ± SD (pmol/mg)	CV (%)	Range (pmol/mg)
CYP2A6	Control neonate	0.17	0.60 ± 1.34	223	0.03–5.01
	BA neonate****	6.75	10.6 ± 9.75	92.4	0.56–34.9
	Control infant	0.29	0.47 ± 0.51	107.6	0.09–1.44
	BA infant***	8.95	9.57 ± 5.40	56.4	2.07–19.9
CYP2B6	Control neonate	0.51	0.65 ± 0.86	132	0.04–3.36
	BA neonate***	1.75	4.46 ± 5.95	133	0.34–19.1
	Control infant	0.86	1.70 ± 2.02	118.3	0.33–5.70
	BA infant	3.29	2.76 ± 1.67	60.4	0.47–5.28
CYP2C8	Control neonate	0.58	1.12 ± 1.78	159	0.15–6.86
	BA neonate***	2.41	3.83 ± 3.29	85.9	1.07–12.1
	Control infant	4.39	5.75 ± 4.82	83.8	1.92–14.9
	BA infant	6.63	10.2 ± 8.97	88.1	0.98–30.4
CYP2C9* (rs3758581)	Control neonate	0	0	0	0
	BA neonate	0.27	0.82 ± 1.58	193	0.03–5.70
	Control infant	0	0	0	0
	BA infant	0.11	0.77 ± 2.06	268.2	0–7.29
CYP2C9	Control neonate	6.64	14.4 ± 18.0	125	1.69–65.8
	BA neonate***	60.6	70.6 ± 46.4	65.7	5.80–152.8
	Control infant	22.6	25.5 ± 17.8	69.9	10.0–58.6
	BA infant**	60.7	80.1 ± 54.4	67.9	31.7–228.9
CYP2C18	Control neonate	0.06	0.12 ± 0.15	125.3	0–0.54
	BA neonate***	0.57	0.70 ± 0.60	86.1	0–2.20
	Control infant	0.47	0.91 ± 1.25	136.5	0.01–3.29
	BA infant	0.39	0.86 ± 1.01	117.6	0.09–3.27
CYP2C19	Control neonate	0.63	1.21 ± 1.40	116.2	0.08–4.52
	BA neonate	1.77	1.84 ± 1.43	77.8	0.15–5.28
	Control infant	4.62	8.25 ± 8.49	102.9	2.07–23.4
	BA infant*	0.88	2.06 ± 2.29	111	0.25–7.24
CYP2E1	Control neonate	0.88	2.13 ± 2.96	139	0.15–10.8
	BA neonate****	13.3	14.3 ± 6.13	42.9	0.91–26.5
	Control infant	4.34	6.91 ± 6.81	98.5	0.85–18.3
	BA infant*	14.1	15.7 ± 6.17	39.4	7.67–28.4
CYP2J2	Control neonate	0.02	0.03 ± 0.02	82.2	0–0.07
	BA neonate****	0.62	0.69 ± 0.48	68.5	0.02–1.85
	Control infant	0.01	0.01 ± 0.01	109.5	0–0.01
	BA infant***	0.43	0.44 ± 0.23	52.1	0.09–0.86
CYP3A4	Control neonate	2.98	6.36 ± 8.24	129.5	0.49–29.5
	BA neonate**	16.1	29.5 ± 38.3	129.9	0.66–135.1
	Control infant	10.5	31.9 ± 47.9	150	4.38–126.9
	BA infant	16.3	22.4 ± 16.1	71.9	5.40–55.5
CYP3A5	Control neonate	0.28	0.49 ± 0.51	103.5	0.1–2.0
	BA neonate**	0.98	2.04 ± 3.02	147.6	0.16–11.4
	Control infant	0.98	2.21 ± 3.37	152.4	0.19–8.99
	BA infant	0.83	4.15 ± 6.78	163.5	0.46–22.8
CYP3A7	Control neonate	49.1	41.6 ± 24.1	58.1	5.73–74.2
	BA neonate	58.9	65.8 ± 33.0	50.2	28.0–141.9
	Control infant	39.4	46.6 ± 27.6	59.3	22.3–99.7
	BA infant	62.2	63.1 ± 37.8	59.9	15.9–157.4
CYP4A11	Control neonate	1.62	2.21 ± 1.61	72.8	0.37–4.99
	BA neonate	2.57	3.38 ± 2.0	59.3	0.04–6.62
	Control infant	1.93	1.67 ± 0.68	40.4	0.55–2.35
	BA infant**	3.79	3.96 ± 1.48	37.4	1.70–6.67
CYP4A22	Control neonate	0.16	0.31 ± 0.30	97.5	0.04–0.94
	BA neonate	0.38	0.66 ± 0.70	106.5	0.03–2.69
	Control infant	0.29	0.34 ± 0.21	62.8	0.10–0.60
	BA infant	0.28	0.41 ± 0.36	88.5	0–1.20
CYP4F2	Control neonate	0.07	0.19 ± 0.28	145.7	0–0.96
	BA neonate****	1.31	1.62 ± 1.09	67.7	0.02–3.23
	Control infant	0.11	0.28 ± 0.43	152	0–1.11
	BA infant***	1.89	2.28 ± 1.85	81.0	0.41–7.07
CYP4F3	Control neonate	1.46	1.97 ± 1.47	74.5	0.26–4.92
	BA neonate**	4.71	4.96 ± 2.53	51.0	0.16–9.35
	Control infant	1.91	2.19 ± 1.03	46.8	1.27–3.42
	BA infant*	4.66	4.39 ± 1.74	39.7	1.87–6.58
CYP4F11	Control neonate	3.27	3.88 ± 2.08	53.6	0.87–7.83
	BA neonate*	1.14	1.74 ± 1.58	90.5	0.17–4.55
	Control infant	5.65	6.06 ± 2.16	35.6	2.76–8.80
	BA infant**	1.98	2.05 ± 1.06	51.5	0.31–3.60
CYP4F12	Control neonate	0	0	0	0
	BA neonate	0.12	0.14 ± 0.09	69.2	0.01–0.38
	Control infant	0	0	0	0
	BA infant	0.10	0.12 ± 0.04	34.1	0.07–0.19
CYP4V2	Control neonate	0.06	0.10 ± 0.08	83.5	0.01–0.26
	BA neonate****	0.44	0.41 ± 0.16	39.2	0.17–0.69
	Control infant	0.09	0.18 ± 0.22	122.7	0.04–0.61
	BA infant*	0.45	0.48 ± 0.20	41.0	0.26–0.98
CYP7B1	Control neonate	0.01	0.02 ± 0.03	152.2	0–0.13
	BA neonate****	0.25	0.28 ± 0.28	102.6	0.02–1.18
	Control infant	0.01	0.01 ± 0.01	77.5	0–0.01
	BA infant***	0.24	0.29 ± 0.21	74.9	0.09–0.85
CYP8B1	Control neonate	1.68	2.26 ± 1.97	87.5	0.37–7.48
	BA neonate	2.94	2.88 ± 1.12	38.7	0.56–4.51
	Control infant	1.60	2.34 ± 2.19	93.8	0.87–6.76
	BA infant	2.43	2.35 ± 0.75	31.8	1.35–3.71
CYP20A1	Control neonate	1.25	1.46 ± 0.62	42.8	0.34–2.48
	BA neonate**	0.72	0.75 ± 0.35	47.2	0.04–1.46
	Control infant	1.88	1.87 ± 0.65	34.6	1.12–2.94
	BA infant**	0.61	0.76 ± 0.47	61.7	0.25–1.71
CYP27A1	Control neonate	0.82	0.97 ± 0.80	82.1	0.23–2.94
	BA neonate****	5.12	5.07 ± 1.98	39.2	0.54–8.01
	Control infant	0.64	0.69 ± 0.22	32.1	0.42–1.06
	BA infant***	5.59	5.73 ± 1.44	25.1	3.10–8.29
CYP39A1	Control neonate	0.16	0.17 ± 0.13	72.9	0.02–0.39
	BA neonate	0	0	0	0
	Control infant	0.26	0.23 ± 0.08	32.9	0.09–0.29
	BA infant	0	0	0	0

Data are presented from 13 neonatal control livers, 13 neonatal BA livers, 6 infant control livers, and 12 infant BA livers. Data include the median, mean ± standard deviation (SD), % coefficient of variation (CV), and range of abundance. Statistical significance is indicated by asterisks; *P < 0.05; **P < 0.01; ***P < 0.001; ****P < 0.0001 vs. corresponding control group (Mann–Whitney *U* test).

### Differential CYP enzyme expression in control and BA livers from infants

CYP2A6, CYP2C8, CYP2C9, and CYP2C9* (rs3758581) showed higher expression in infant BA livers, with average levels increased across these enzymes compared with control (**Figure** **1** **b**). For instance, CYP2A6 expression rose from a range of 0.1–1.4 pmol/mg protein in controls to 2.1–19.9 pmol/mg protein in BA livers, while CYP2C9 exhibited a substantial increase from 10.0 to 58.6 pmol/mg protein in controls to 31.7–229 pmol/mg protein in BA livers. This increase is accompanied by a reduction in variability for most enzymes, as indicated by the decreased CV (**Table** **1**), suggesting more consistent expression patterns in infant BA livers. Conversely, CYP2C19 exhibited a marked reduction, with mean expression levels dropping from 8.25 ± 8.49 pmol/mg protein in controls to 2.1 ± 2.29 pmol/mg protein in BA livers (**Figure** **1** **b**, **Table** **1**). CYP4F11 and CYP3A4 showed similar expression across the two conditions. These findings indicate that BA may lead to increased expression of specific CYP enzymes, while suppressing others.

### Expression of UDP‐glucuronosyltransferase (UGT) enzymes in control and BA livers from neonates and infants

**Figure 2:**
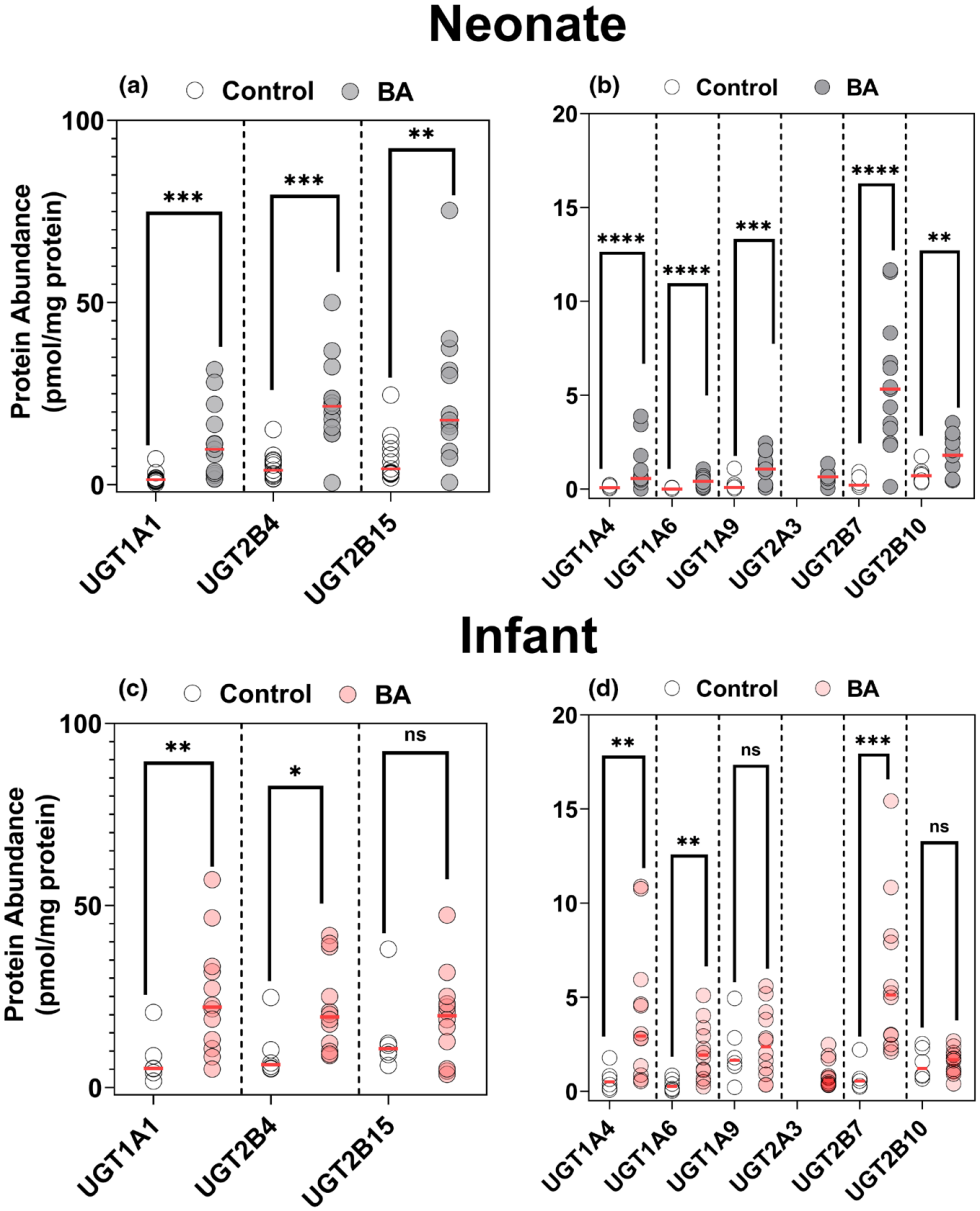
Protein expression UGT enzymes in biliary atresia (BA) and control livers from neonates (a, b) and infants (c, d). Statistical significance is indicated by asterisks (*P < 0.05, **P < 0.01, ***P < 0.001, ****P < 0.0001), and “ns” denotes non‐significant differences. These data demonstrate a significant increase of several UGT enzymes in BA groups compared to controls in both neonatal and infant livers. Horizontal red lines represent the median. Protein abundance is expressed in pmol/mg protein.

Analysis of UGT enzyme expression yielded similar results to those observed for CYP enzymes (**Figure** **2**), with significantly higher levels in BA livers compared with their respective control groups. For example, the mean expression of UGT1A1 was approximately six‐fold higher in BA neonates and three‐fold higher in BA infants. Similarly, UGT1A6 showed a 30‐fold and 6‐fold higher expression in BA neonates and BA infants, respectively (**Table** **S3**, **Figure** **2**). UGT2B7 expression was 16‐fold higher in BA neonates and 8‐fold higher in BA infants, while UGT1A4 exhibited an 11‐fold and 6‐fold higher expression in neonates and infants, respectively. CVs were generally lower in BA neonates for enzymes, such as UGT1A1 and UGT2B7 (**Table** **S3**), indicating more consistent expression, although some enzymes, including UGT2B4, exhibited higher variability (CV = 75% in disease vs. 53% in controls). Other UGT enzymes showed moderate changes, with mean expression levels approximately two–four‐fold higher in the BA group compared with infant controls. These results highlight consistently higher UGT expression in response to BA.

### Comparison of transporter protein expression between disease and control groups

**Figure 3:**
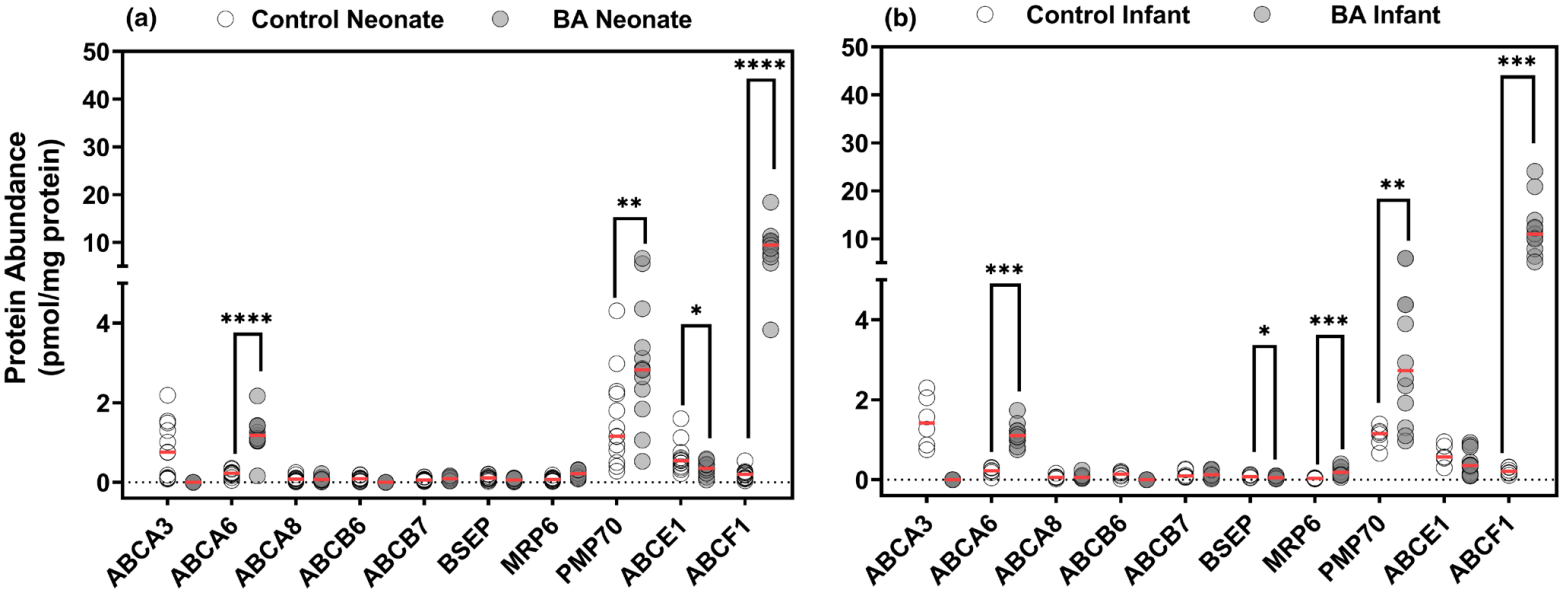
Protein abundance of ABC transporters in neonatal and infant livers under control and biliary atresia (BA) conditions. Comparison of ABC transporters in (a) neonatal liver controls (open circles) and BA livers (closed circles), and in (b) infant liver controls (open circles) and BA livers (closed circles). The horizontal red lines reflect median values. Statistical significance is indicated by asterisks (*P < 0.05, **P < 0.01, ***P < 0.001, ****P < 0.0001). Protein abundance is expressed in pmol/mg protein.

**Figure 4:**
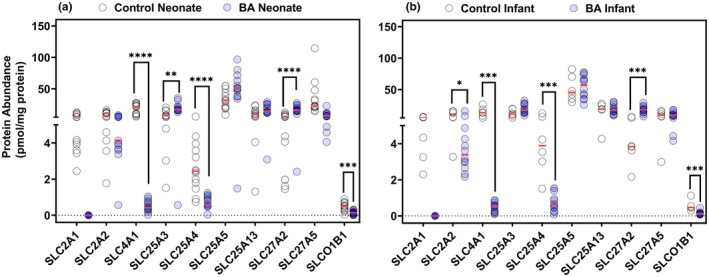
Protein abundance of SLCs in neonatal and infant livers under control and biliary atresia (BA) conditions. Comparison of SLC transporters in (a) neonatal liver controls (open circles) and BA livers (closed circles), and in (b) infant liver controls (open circles) and BA livers (closed circles). The horizontal red lines reflect median values. Statistical significance is indicated by asterisks (*P < 0.05, **P < 0.01, ***P < 0.001, ****P < 0.0001). Protein abundance is expressed in pmol/mg protein.

The expression of 10 ABC transporters (**Figure** **3**, **Table** **2**) and 10 SLCs (**Figure** **4**) was examined in this study. The majority of these proteins were of low abundance. ABCA6 and ABCF1 showed higher expression in both biliary atresia age groups, 5‐fold and > 50‐fold respectively, suggesting a consistent pattern across developmental stages. ABCA3 showed no expression in either of the two biliary atresia groups. Moreover, no differences were observed in ABCA8, ABCB6, and ABCB7 expression data between age groups. These findings highlight significant increases in the expression of specific ABC transporters, particularly ABCF1 and PMP70 (ABCD3), in BA conditions for both groups, reflecting adaptive changes in response to the disease. PMP70 expression showed marked inter‐individual variability across most groups. In neonatal samples, the CV was 74% in controls and 55% in biliary atresia, whereas in infant biliary atresia samples variability remained high (CV 56%) (**Table** **2**). In contrast, infant control livers exhibited substantially lower variability, with a CV of 23%, indicating more uniform PMP70 expression within this group. Despite this variability, mean PMP70 expression was increased in biliary atresia compared with controls in both neonatal and infant cohorts (**Table** **2**). The expression of SLCs (**Figure** **4**, **Table** **S4**) in neonatal livers demonstrated considerable variability across targets, with mean expression ranging from 0.47 pmol/mg protein (SLCO1B1/SLC21A6) to 33 pmol/mg protein (ADT2/SLC25A5) in controls (**Figure** **4** **a**). CV values highlighted the variability, spanning from 45% (ADT2/SLC25A5) to 79% (S27A5/SLC27A5) (**Figure** **4** **a**). When comparing fold differences between controls and BA, B3AT/SLC4A1, ADT1/SLC25A4, and S27A5/SLC27A5 showed notable reductions in BA (34‐fold, 4‐fold, and 3.8‐fold, respectively), while most other transporters exhibited minimal differences (**Figure** **4** **a**). This indicates that while the general trends in transporter alterations are consistent, the extent of these differences may be age‐dependent, reflecting developmental changes, or disease progression over time.

**Table 2:** Abundance of ABC transporters in healthy and BA livers from neonates and infants

Transporter	Donors	Median (pmol/mg)	Mean ± SD (pmol/mg)	CV (%)	Range (pmol/mg)
ABCA3	Control neonate	0.76	0.86 ± 0.71	83.1	0.08–2.19
	BA neonate	0	0	0	0
	Control infant	1.42	1.47 ± 0.63	42.6	0.75–2.30
	BA infant	0	0	0	0
ABCA6	Control neonate	0.23	0.21 ± 0.09	44.0	0.05–0.35
	BA neonate****	1.18	1.21 ± 0.44	36.2	0.16–2.17
	Control infant	0.23	0.21 ± 0.09	43.6	0.05–0.31
	BA infant***	1.11	1.13 ± 0.26	23.2	0.75–1.74
ABCA8	Control neonate	0.08	0.09 ± 0.07	79	0.01–0.25
	BA neonate	0.07	0.08 ± 0.06	71.9	0.01–0.22
	Control infant	0.06	0.07 ± 0.05	67.6	0.02–0.16
	BA infant	0.06	0.07 ± 0.06	77.5	0.03–0.24
ABCB6	Control neonate	0.09	0.10 ± 0.06	58.2	0.01–0.19
	BA neonate	0	0	0	0
	Control infant	0.14	0.13 ± 0.07	52.9	0.02–0.21
	BA infant	0	0	0	0
ABCB7	Control neonate	0.06	0.07 ± 0.04	53.6	0.02–0.14
	BA neonate	0.09	0.09 ± 0.04	41.3	0.03–0.16
	Control infant	0.10	0.14 ± 0.09	67.6	0.06–0.27
	BA infant	0.13	0.13 ± 0.08	64.2	0.02–0.26
BSEP	Control neonate	0.11	0.11 ± 0.05	48.4	0.02–0.21
	BA neonate*	0.06	0.06 ± 0.03	52.6	0.01–0.11
	Control infant	0.08	0.09 ± 0.03	39.9	0.05–0.13
	BA infant*	0.05	0.05 ± 0.03	49.2	0.02–0.10
MRP6	Control neonate	0.07	0.07 ± 0.04	60.8	0.02–0.18
	BA neonate****	0.22	0.21 ± 0.07	34.2	0.08–0.32
	Control infant	0.04	0.03 ± 0.01	36.3	0.02–0.05
	BA infant***	0.19	0.20 ± 0.10	49.0	0.06–0.40
PMP70	Control neonate	1.16	1.56 ± 1.16	74.3	0.28–4.31
	BA neonate**	2.83	3.08 ± 1.68	54.6	0.53–6.70
	Control infant	1.16	1.09 ± 0.25	23.4	0.66–1.39
	BA infant**	2.73	3.14 ± 1.76	56.1	0.97–5.94
ABCE1	Control neonate	0.54	0.62 ± 0.37	59.9	0.22–1.60
	BA neonate*	0.35	0.35 ± 0.15	44.3	0.06–0.59
	Control infant	0.57	0.63 ± 0.23	37	0.30–0.95
	BA infant	0.36	0.41 ± 0.30	72.7	0.09–0.93
ABCF1	Control neonate	0.20	0.20 ± 0.12	62.6	0.04–0.54
	BA neonate****	9.43	9.32 ± 3.44	36.9	3.83–18.4
	Control infant	0.21	0.22 ± 0.08	38.5	0.11–0.32
	BA infant***	11	12.1 ± 5.50	45.5	5.19–24.1

Data include the median, mean ± standard deviation (SD), % coefficient of variation (CV), and range of abundance. Statistical significance is indicated by asterisks; *P < 0.05; **P < 0.01; ***P < 0.001; ****P < 0.0001 vs. corresponding control group (Mann–Whitney *U* test).

### Relative differences in protein abundance of enzymes and transporters in neonate and infant livers with biliary atresia compared with controls

**Figure 5:**
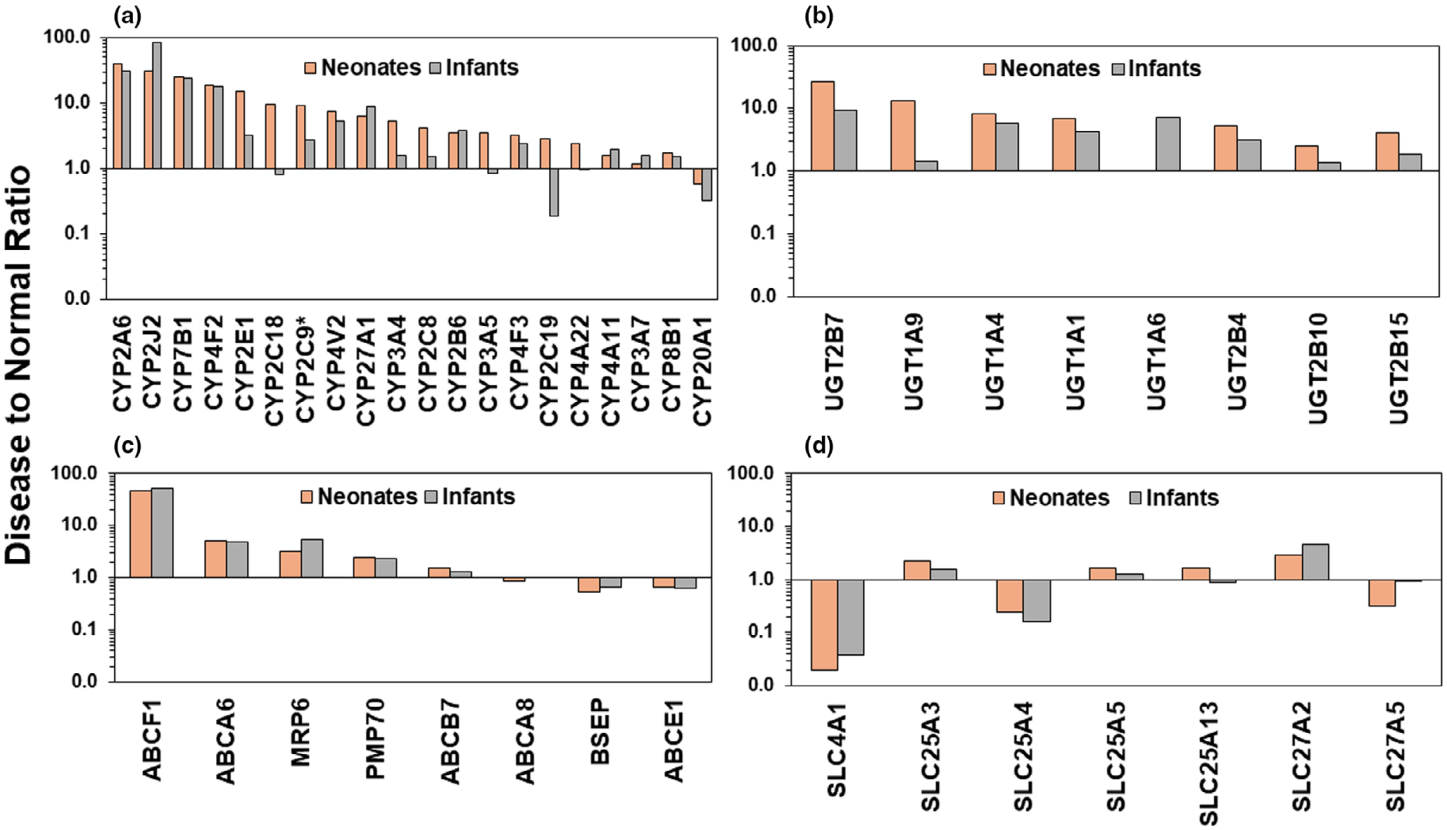
Relative differences in median protein abundance in neonates and infants with biliary atresia (BA) compared with controls for (a) CYP enzymes (b) UGT enzymes (c) ABC transporters, and (d) solute carriers. Orange bars = neonates with BA relative to control neonates; gray bars = infants with BA relative to control infants. Values > 1 indicate higher protein abundance in BA livers; values < 1 indicate reduced abundance.

Relative differences in median protein abundance in neonates and infants with biliary atresia relative to their respective controls are shown as fold change data in **Figure** **5**. The most pronounced changes were observed in CYP enzymes, where neonates consistently showed higher disease‐to‐healthy ratios than infants. CYP2A6, CYP2J2, CYP2E1, and CYP7B1 demonstrated the highest expression difference in neonates, with fold changes exceeding 15‐fold compared with controls. Most CYP enzymes (CYP3A4, CYP2C8, and CYP2E1) showed moderate to high expression changes in neonates, while infants generally exhibited lower expression for the same enzymes. In **Figure** **5** **a**, median expression of CYP2C18, CYP2C19, CYP3A5, and CYP20A1 in infants with BA was consistently lower than controls. This indicates generally low expression ratios in infants with BA, contrasting with the pattern seen in neonates, where CYP enzyme levels (except CYP20A1) were predominantly elevated above control levels. UGT enzymes displayed variable expression patterns between age groups (**Figure** **5** **b**). UGT2B7 showed higher expression in BA in neonates and infants relative to control, and the effect was more pronounced in neonates. Similar trends were seen for UGT1A4 and UGT1A1. **Figure** **5** **c** illustrates fold change data for various ABC transporters in neonates and infants with BA compared with controls. The data demonstrate higher expression of several ABC transporters, particularly in neonates. ABCF1 and ABCA6 show the highest fold changes, with neonates exhibiting disease‐to‐normal ratios approaching or exceeding 10, while infants displayed more moderate elevations. Other transporters, such as MRP6 and PMP70, also showed higher expression in neonates compared with controls. In contrast, BSEP and ABCE1 demonstrated ratios close to or below one in both groups, indicating little change or slight reduction in expression relative to controls. Overall, these findings suggest that ABC transporter expression is more prominent in neonates than in infants with biliary atresia, highlighting age‐related differences in hepatic transporters. SLC transporter proteins demonstrated more heterogeneous expression patterns, and SLC4A1, SLC25A4, and SLC27A5 exhibited varying degrees of low expression.

### Distribution of CYP and UGT enzymes in neonatal and infant livers in BA livers and controls

Pie charts in **Figure** **S1** show that CYP enzyme distribution varied notably across age groups and disease. CYP3A7 remained dominant in neonates, while CYP3A4 increased in infants. BA conditions altered the relative abundance of several CYP isoforms. Detailed data and interpretation are provided in **Supplementary Information**. UGT2B15, UGT2B4, and UGT1A1 were the predominant isoforms across all groups (**Figure** **S2**). BA‐related changes included a reduction in UGT2B15 and increased proportions of UGT2B7 and the polymorphic UGT2B15 ^D85Y^ variant. Full details are presented in **Supporting Methods**.

### Distribution of ABC transporters and SLCs in neonatal and infant livers in BA livers and controls

BA conditions led to a marked increase in ABCF1 expression and a concurrent reduction in PMF70 (ABCD3), altering the transporter profile in both neonatal and infant livers (**Figure** **S3**). Developmental differences were also observed between neonatal and infant control groups. SLC25A5 emerged as the dominant transporter in both neonatal and infant BA livers, with disease‐ and age‐related shifts in SLC expression patterns (**Figure** **S4**). More information is available in **Supporting Methods**.

## DISCUSSION

The study provides, for the first time, an indication of changes in hepatic drug‐metabolizing enzymes and transporter proteins in a rare disease using a unique set of neonatal and infant livers from patients with BA. Liver samples from a control group of neonatal and infant livers were processed using the same methodology alongside the BA samples and analyzed in parallel. Therefore, we were able to establish the relative changes in abundance of the target proteins without concerns regarding inter‐laboratory differences reported in LC–MS/MS proteomic literature.^32^ The most significant finding was related to non‐uniformity of the changes in protein expression in BA, where expression relative to controls was higher for some proteins, lower for other proteins, or not different. These changes have direct implications for drug exposure. To illustrate, several CYP enzymes, including CYP2A6, CYP2B6, CYP2E1, and CYP2C9, were markedly increased in BA neonates (up to 17‐fold), contrasting with reduced expression seen in animal models.^33^ Similarly, UGT1A1, UGT2B4, and UGT2B7 showed up to a 16‐fold increase, suggesting enhanced glucuronidation and potential for increased drug clearance. A dramatic 46‐fold increase in ABCF1 and a 3‐fold increase in MRP6, along with reduced expression of SLC transporters (SLC4A1, SLC25A4, SLC27A5), indicate altered drug transport, possibly impacting hepatic drug uptake and efflux. Additionally, enzymes such as CYP4F11, CYP20A1, and CYP2C19 showed reduced expression. These changes in enzyme and transporter expression have direct implications for drug dosing in BA patients: reduced transporter activity may lead to lower intracellular drug concentrations, while downregulation of efflux transporters (e.g., MRP6) could result in intracellular accumulation, potentially enhanced toxicity. Altered enzyme levels can result in unpredictable changes in drug metabolism and clearance. PMP70 is a peroxisomal membrane ABC transporter involved in the import of fatty and bile acid intermediates, and its expression is metabolically responsive rather than constitutive.^34^, ^35^ In biliary atresia, bile acid metabolism, inflammation, and fibrosis are heterogeneously affected between individuals, which is likely to drive variable peroxisomal adaptation and contribute to increased inter‐individual variability in PMP70 expression observed in both neonatal and infant BA groups.^36^ In contrast, infant control livers represent a narrow developmental and metabolic window characterized by tightly regulated hepatic and bile acid metabolism, consistent with the markedly reduced variability observed in this group.^37^, ^38^ Consequently, standard dosing regimens based on healthy or adult reference populations may be inappropriate for BA patients, necessitating careful consideration and, where possible, individualized dose adjustments to ensure therapeutic efficacy and minimize the risk of adverse events.

### Hypothetical implications of biliary atresia in drug dosing

Although this study is based on *in vitro* proteomic measurements, the observed changes in CYP and UGT enzyme abundance have potential clinical relevance. Specifically, alterations in pathway specific enzymes may differentially affect the clearance of drugs commonly used in biliary atresia, rather than indicating a uniform reduction in hepatic metabolism. The use of drugs in pediatrics with BA is generally limited to prophylactic post‐operative treatment and treatment of cholangitis, including antibiotics, such as amoxicillin‐clavulanic acid,^39^, ^40^ and analgesics, such as paracetamol or morphine for pain management. Metabolism of Augmentin, a combination of amoxicillin and clavulanic acid, is limited in the liver. Most amoxicillin is excreted unchanged in urine with hepatic metabolism playing a minimal role in the biotransformation of most penicillins. Clavulanic acid is metabolized by the liver without the involvement of specific CYP enzymes, and only 30–40% is excreted unchanged in the urine.^41^ Thus, dosage adjustment for these drugs is expected to be minimal. However, paracetamol and morphine are primarily metabolized by CYP and UGT enzymes in the liver. Paracetamol is commonly used to treat pain and fever in pediatrics. Dosage adjustment in patients with impaired liver function is typically designed to err on the side of caution because of the potential increased risk of toxicity.^42^

Paracetamol is primarily metabolized by glucuronidation and sulfite conjugation to non‐toxic and inactive metabolites. A small proportion (5–9%) is metabolized by CYP2E1 to N‐acetyl‐p‐benzoquinone imine (NAPQI), a toxic metabolite.^43^ Whilst this is only produced in small amounts, if it is not effectively metabolized further, it can lead to severe liver damage. Glutathione transferase, a detoxification pathway for NAPQI in the liver, is very effective at removing this toxic compound, though once glutathione is depleted, detoxification is reduced.^44^ Studies on the impact of liver‐related diseases and disorders on glutathione abundance have been largely inconclusive. However, malnutrition in these patients increases the risk of glutathione depletion and therefore increases the risk of hepatoxicity.^42^ In the biliary atresia samples examined in this study, all relevant proteins within the liver were much higher in abundance compared to the control populations. This suggests that paracetamol is likely metabolized via the CYP2E1 pathway (in addition to the UGT pathway), potentially leading to increased production and build‐up of NAPQI because of glutathione saturation. This highlights the necessity for paracetamol dosage adjustments in patients with liver disease. Modeling the impact of increased CYP2E1 on NAPQI production and glutathione depletion is crucial for establishing safe and effective pain management strategies.

Morphine is primarily metabolized by UGT2B7 in the liver to two main metabolites. Approximately 57% is metabolized to morphine‐3‐glucuronide (M3G), an inactive metabolite, and 10% to the more pharmacologically active morphine‐6‐glucuronide (M6G).^45^, ^46^ Morphine works by activating the μ‐opioid receptor, with 85% of morphine's analgesic effect being attributed to M6G. In contrast, M3G has been shown to have up to 200 times lower binding affinity compared with morphine and consequently lacks any analgesic effect.^45^ Therefore, an increase in UGT2B7 in the liver could have serious implications. In the first instance, there is likely to be increased M6G production, which may lead to toxic effects; morphine has a higher affinity for the μ2‐opioid receptor than M6G and has been linked to many of the adverse effects from μ‐opioid agonists. These include sedation and respiratory depression.^46^ Reduced doses and/or longer dosing intervals are implemented to account for the increased (approximately double) half‐life of the drug in patients with liver disease. However, as with paracetamol, there are no evidence‐based recommendations for effective dose reduction in such patients.^47^ These are just two examples of medications that are typically used in biliary atresia. In more rare instances, other complications or diseases may arise (orphan disease populations) and treatment with other drugs may be necessary. With such limited information on the impact of the reduced abundance of drug‐metabolizing enzymes and transporter proteins in these populations, successful treatment requires constant patient monitoring and drug dosage adjustment. In silico modeling in pediatric populations could support more informed drug dosing recommendations. The quantitative protein abundance data presented in this study can serve as critical input parameters for pediatric PBPK models^48^, ^49^, ^50^ through the “bottom‐up” approach, where measured protein abundances are used as scaling factors to predict *in vivo* hepatic drug clearance.^48^ The observed increases in CYP and UGT enzymes and alterations in transporter proteins can be directly incorporated into PBPK models to predict altered drug metabolism and transport capacity in BA patients. Quantitative proteomic data have previously been shown to improve pediatric PBPK model performance, particularly for drug transporters^48^, ^49^, and the disease‐specific protein expression profiles reported here address a critical knowledge gap in current pediatric PBPK models that largely rely on healthy populations. Future integration of these proteomic data with *in vitro* functional assays and clinical pharmacokinetic observations in BA patients would further support model‐informed precision dosing in this vulnerable population.

### The effect of disease on protein expression and ontogeny

The pie charts across **Figures** **S1–S4** illustrate proportional shifts in the distribution of CYP and UGT enzymes, as well as ABC and SLC transporters, across different age groups and disease states. These changes represent relative contributions of individual proteins rather than absolute abundance and should be interpreted in the context of fraction metabolized or transported, which are key determinants of the magnitude of drug–drug interactions (DDIs). Protein abundances are reported as pmol/mg total protein and therefore reflect relative enzyme and transporter density within the hepatic proteome rather than absolute whole‐organ expression. Disease‐related changes in liver mass and functional hepatocyte content in biliary atresia may further influence overall hepatic drug‐metabolizing capacity and are not captured by tissue‐normalized measurements alone. A higher proportional presence of an enzyme or transporter does not necessarily translate to increased metabolic or transport capacity, particularly in diseased livers, where overall protein expression may be impaired. Given the limited clinical evidence on DDIs in pediatric populations,^7^, ^51^ especially under pathological conditions, such as BA, these findings should be viewed as hypothesis generating, reinforcing the need for quantitative data, and model‐informed approaches to support safer and more effective drug therapy in neonates and infants. A limitation of this study is that control liver tissue from completely healthy neonates and infants is not available; therefore, control samples were derived from patients with non‐hepatic conditions, such as cardiac dysfunction. While there is limited evidence that cardiac disease directly alters hepatic enzymes or transporters in this age group, this should be considered when interpreting the data. Best practices in drug dosing recommendations for neonates have recently been reviewed.^10^ The absence of clinical data in certain sub‐groups led the authors to consider modeling and simulation as a potential alternative when specific pharmacokinetic data are absent. This study provides information on protein abundance differences for drug‐metabolizing enzymes and transporters in neonates and infants with BA vs. control. Since the relative changes are not uniform, the drug dosage adjustment cannot be done uniformly and will most likely be dependent on each drug and their specific routes of elimination to avoid increased risk of toxicity and/or ineffective treatment.

## CONCLUSION

Designing drug dosing regimens for neonates and infants already poses many challenges and risks. The added complication of disease, such as BA and its effect on drug metabolism as part of the disease process, further complicates this challenge. This study is one of the first of its kind to establish the systems parameters for hepatic drug handling in BA. These can be used in the development of mechanistic *in silico* models under PBPK framework to enhance drug dosing regimens in this patient population.

The use of *in silico* tools provides much better (evidence‐based) recommendations than the variable guesswork by healthcare providers when there is no clinical evidence available. The currently available PBPK platforms can account for numerous systems factors that might be different in a target population,^52^ however, their interface is not designed for clinical use. Although this report provides some of the systems information with implications for hepatic drug handling, as Darwich et al. have indicated in their white paper,^53^ there are other elements that need to be fulfilled before model‐informed precision dosing becomes the norm for these special groups.

## Funding

This research was supported by Children's Liver Disease Foundation (CLDF), Birmingham, West Midlands, UK.

## Conflict of interest

Amin Rostami‐Hodjegan is an employee of Certara. All other authors declared no competing interests for this work.

## Author contributions

Z.M‐Al., M.H., B.A., J.B., N.A., A.R‐H wrote the manuscript; Z.M‐Al., B.A., J.B., N.A., and A.R‐H. designed the research; M.H. performed the research; Z.M‐Al. and J.B. analyzed the data.

## Supporting information


Data S1.
