# Supplementary material for: Unveiling Hepatic Protein Alterations in Neonatal and Infant Biliary Atresia
Source: Clin Pharmacol Ther. 2026 Mar 4;119(6):1584–96. doi: 10.1002/cpt.70244 (PMC13156361; doi:10.1002/cpt.70244)
Supplement: Supplementary file 1 — Data S1. [file CPT-119-1584-s001.docx]

**Unveiling Hepatic Protein Alterations in Neonatal and Infant Biliary Atresia**

**Zubida M. Al-Majdoub*, Martyn Howard, Brahim Achour, Jill Barber, Naved Alizai, Amin Rostami-Hodjegan**

*Correspondence to: Dr Z Al-Majdoub; email: [zubida.al-majdoub@manchester.ac.uk](mailto:zubida.al-majdoub@manchester.ac.uk)

**Supporting Information Content**

**Supporting Methods**

**Supporting Results**

**Table S1** Demographic data of control liver samples from neonates, infants, and one child.

**Table S2** Demographic data of biliary atresia liver samples.

**Table S3** Abundance of UDP-glucuronosyltransferase (UGT) enzymes in healthy and BA livers from neonates and infants.

**Table S4**: Abundance of SLC transporters in healthy and BA livers from neonates and infants.

**Figure S1** Distribution of CYP enzyme isoforms in neonatal and infant livers under control and BA conditions.

**Figure S2** Pie charts representing the proportional distribution (%) of various UDP-glucuronosyltransferase (UGT) isoforms in livers of control neonates, neonates with biliary atresia (BA), control infants, and infants with BA.

**Figure S3** Pie charts showing the proportional distribution (%) of ABC transporters in control neonates, BA neonates, control infants, and BA infants. Each segment represents a transporter’s relative contribution within the group.

**Figure S4** Pie charts showing the proportional distribution (%) of SLC transporters in four sample groups: control neonates, BA neonates, control infants, and BA infants.

**Table S1:** Demographic data of control liver samples from neonates, infants, and one child

| **ID** | **Age (Years)** | **Age (Months)** | **Age (Weeks)** | **Age (Days)** | **Ethnicity** | **Population** | **Sex** | **Diagnosis** | **Cause of death** |
| --- | --- | --- | --- | --- | --- | --- | --- | --- | --- |
| N12 |  |  |  | 1 | CA | Neonates | F | Decompensatio cordis | Asphyxia |
| N21 |  |  |  | 1 | CA | Neonates | M | Chromosomal abnormality |  |
| N16 |  |  |  | 2 | CA | Neonates | M | Sepsis |  |
| N22 |  |  |  | 2 | CA | Neonates | F | Necrotizing enterocolitis | Respiratory insufficiency |
| N15 |  |  |  | 3 | CA | Neonates | F | Aspiration meconium |  |
| N1 |  |  |  | 4 | CA | Neonates | F | Hemangioendothelioma | Cardio-pulmonary failure |
| N18 |  |  | 1 | 0 | CA | Neonates | M | Necrotizing enterocolitis | Acute circulatory failure |
| N10 |  |  | 1 | 2 | CA | Neonates | F | Hepes simplex infectie |  |
| N9 |  |  | 1 | 5 | CA | Neonates | F | Oligomeganephrony, congential abnormality | Respiratory insufficiency |
| N8 |  |  | 3 | 2 | CA | Neonates | F | Sepsis | Sepsis E Coli encephalitis |
| N23 |  |  | 3 | 3 | CA | Neonates | F | Unknown, possibly facialisparesis | Respiratory insufficiency, bradycardia |
| N13 |  |  | 4 | 1 | CA | Neonates | F | Intracranial bleeding |  |
| N 7 |  | 1 | 0 | 0 | CA | Neonates |  |  |  |
| N19 |  |  | 5.86 |  | CA | Infants | F | Sudden infant death syndrome |  |
| N11 |  |  | 8.29 |  | CA | Infants | M | Abortus provocatus, trisomie | Viral encephalopathy and respiratory insufficiency |
| N17 |  |  | 10.57 |  | CA | Infants | M | Decompensatio cordis | Acute circulatory failure |
| N 20 | 1 |  | 8 |  | CA | Infants | F | Bronchopneumonia and congenital cardiac abnormality (AVSD) | Cardiac abnormality |
| N14 | 1 |  | 14.86 |  | CA | Infants | M | Cardiomyopathy | Brain infarct during LVAD procedure |
| N24 | 7 |  |  |  | CA | Children | F | Myocarditis, bronchopneumonia | Respiratory and circulatory insufficiency |

CA= Caucasian; F=Female; M= Male

**Table S2:** Demographic data of biliary atresia liver samples

| **ID** | **Age (Months)** | **Age (Weeks)** | **Age (Days)** | **Population** | **Sex** | **Ethnicity** | **Diagnosis** |
| --- | --- | --- | --- | --- | --- | --- | --- |
| 4416 |  | 2 |  | Neonates | F | CA | BA |
| 6313 |  | 2 |  | Neonates | M | CA | BA |
| 5664 |  |  | 15 | Neonates | M | P | BA |
| 7255 |  |  | 20 | Neonates | F | CA | BA |
| 5220 |  | 3 |  | Neonates | M | CA | BA |
| 6314 |  | 3 |  | Neonates | M | Ind | BA |
| 9060 |  | 3 |  | Neonates | F | CA | BA |
| 5667 | 1 |  |  | Neonates | F | CA | BA |
| 6309 | 1 |  |  | Neonates | F | CA | BA |
| 6303 | 1 |  |  | Neonates | M | Ch | BA |
| 6973 | 1 |  |  | Neonates | F | CA | BA |
| 4410 | 1 |  |  | Neonates | F | CA | BA |
| 7258 | 1 |  |  | Neonates | F | P | BA |
| 6979 | 3 |  |  | Infants | M | AS | BA |
| 4409 | 2 |  |  | Infants | F | - | BA |
| 4406 | 2 |  |  | Infants | F | CA | BA |
| 4417 | 2 |  |  | Infants | F | CA | BA |
| 5223 | 2 |  |  | Infants | M | CA | BA |
| 5662 | 2 |  |  | Infants | M | CA | BA |
| 9065 | 2 |  |  | Infants | F | CA | BA |
| 3857 |  | 8 |  | Infants | F | P | BA |
| 4403 |  | 8 |  | Infants | M | P | BA |
| 4415 |  | 8 |  | Infants | M | AF | BA |
| 5225 | 3 |  |  | Infants | M | EUR | BA |
| 7250 | 5 |  |  | Infants | M | CA | BA |

CA= Caucasian; P= Pakistani; AF= African; Ind= Indian; Ch= Chinese; AS= Asian; EUR= European; F=Female; M=Male; BA= Biliary Atresia; - = no information available

**Supporting Methods**

#

# Human liver samples and tissue fractionation

Microsomal fractions were prepared from human liver tissue. Liver tissue was homogenised in homogenisation buffer (1 mM EDTA, 10 mM HEPES, 150 mM KCl, 1 mM DTT, 0.2 mM 4-(2-aminoethyl) benzenesulfonyl fluoride hydrochloride (Pefabloc®), 1 cOmplete™ mini protease inhibitor cocktail tablet (Roche Applied Sciences, Mannheim, Germany, pH 7.4), 10 mL/g of tissue, and the microsomal fraction was extracted using differential centrifugation.^1, 2^ The microsomal pellet was resuspended in storage buffer (0.26 M potassium phosphate, 30% v/v glycerol, pH 7.25), 1 mL/mg tissue, and stored at -80^o^C. Protein content of microsomal fractions was estimated using Pierce™ BCA protein assay kit (ThermoFischer Scientific, Hemel Hempstead, UK) according to the manufacturer’s guidelines. Bovine serum albumin (BSA) was used as a standard and analysis was carried out in triplicates.

**Sample preparation for proteomics**

Solubilisation and reduction of each microsomal fraction was achieved using sodium deoxycholate (final concentration 10% w/v) and DTT (final concentration 0.1 M), incubated at room temperature for 10 min, followed by 30 min at 56 ^o^C. Protein digestion was carried out using the filter-aided sample preparation (FASP) method, as described in our previous publications.^3, 4^ Briefly, Amicon Ultra 0.5 mL centrifugal filters with a 10 kDa molecular weight cut-off (Merck Millipore, Nottingham, UK) were prepared by washing with 200 µL 0.1 M Tris-HCl pH 8.5 and centrifuging at 13,000 g for 15 min. The solubilised and reduced microsomal samples were then transferred to the washed filter units and centrifuged at 14,000 g. Next, 200 µL 8 M urea in 0.1 M Tris-HCl solution was added to the filter units and centrifuged at 14,000 g. Alkylation was performed by adding 50 mM iodoacetamide (IAA) and incubating the samples in the dark for 30 min at room temperature, followed by centrifugation for 10 min at 14,000 g. Buffer exchange was performed using successive washes with 8 M urea in 0.1 M Tris-HCl, followed by 1 M urea in 50 mM ammonium bicarbonate. Protein digestion was achieved through sequential enzymatic digestion^5, 6^ using endopeptidase Lys-C (Wako, Japan) and trypsin (Roche). Lys-C was added at a 1:50 enzyme-to-protein ratio and incubated for 4 h at 30^o^C, followed by the addition of trypsin at a 1:25 ratio and incubation at 37^o^C for overnight. Peptides were collected by centrifugation at 14,000 g for 20 min and eluted with 0.5 M NaCl. The eluted peptides were dried in a vacuum concentrator, resuspended in 50 mM ammonium bicarbonate (pH 8.0), and desalted using a Pierce® C18 column (Thermo Fisher Scientific, Rockford, IL). Finally, the peptides were dried and stored at -80°C until mass spectrometric analysis.

# Liquid chromatography and tandem mass spectrometry (LC-MS/MS)

Dried peptide samples were resuspended in loading buffer (5% v/v acetonitrile in water with 0.1% v/v trifluoroacetic acid). 1 µL of each sample loaded on an Ultimate® 3000 rapid separation liquid chromatography (Dionex Corporation, Sunnyvalle, CA) coupled to an online QExactive HF Hybrid Quadropole-Orbitrap mass spectrometer (ThermoFischer Scientific, Bremen, Germany).

**LC-MS/MS data analysis**

Data analysis was carried out in Progenesis v4.0 (Nonlinear Dynamics, Newcastle-upon-Tyne, UK). The data were exported as Mascot generic files (mgf) and peptides identified and initially assigned to proteins by Mascot (Matrix Science, London, UK). In Mascot, cysteine carbamidomethylation was considered as a fixed modification, and methionine oxidation was considered as a variable modification. The precursor mass tolerance was set to 2 ppm, fragment mass tolerance was set to 0.02 Da, and Trypsin/P was set as the proteolytic enzyme, allowing for one missed cleavage. The list of identified peptides and proteins was imported back into Progenesis for review and the resultant peptide ion files exported as comma-separated values (csv) files.

**Protein identification and quantification**

The results obtained from Mascot were analyzed using a customised database based on the Uniprot Human Protein fasta file (<https://www.uniprot.org/proteomes/UP000005640>). This database contains data from 21,520 proteins and is termed CAPKR11. A razor was created as described in our previous publication.^7^​ The total approach (TPA) was then used to quantify proteins based on the ratio of individual protein MS signal intensity to total proteome MS signal intensity in the sample.

**Supporting Results**

**Distribution of CYP enzyme in neonatal and infant livers under control and biliary atresia (BA) conditions**

The distribution of CYP enzymes revealed noticeable differences in proportional protein abundance across age groups and conditions. In control neonates (Figure S1a), CYP3A7 accounted for 50.6% of expressed CYPs, followed by CYP2C9 (17.5%), and CYP3A4 (7.8%), with the remaining CYPs contributing less than 10% each. However, in control infants (Figure S1c), the proportion of CYP3A7 decreased to 31.4% and other previously low abundant proteins either increased in proportion, such as CYP3A4, which rose from 7.8% to 21.5%, or remained the same, such as the case of CYP2C9. In the BA neonate group and infant groups (Figure S1b, d), the proportions diverged from those of the control neonates. CYP3A7 remained highly expressed in both BA groups, accounting for 26.5-29% of the total CYPs. Notably, CYP2C9 doubled in abundance in the BA groups, while only incremental changes were observed for the remaining proteins.

**
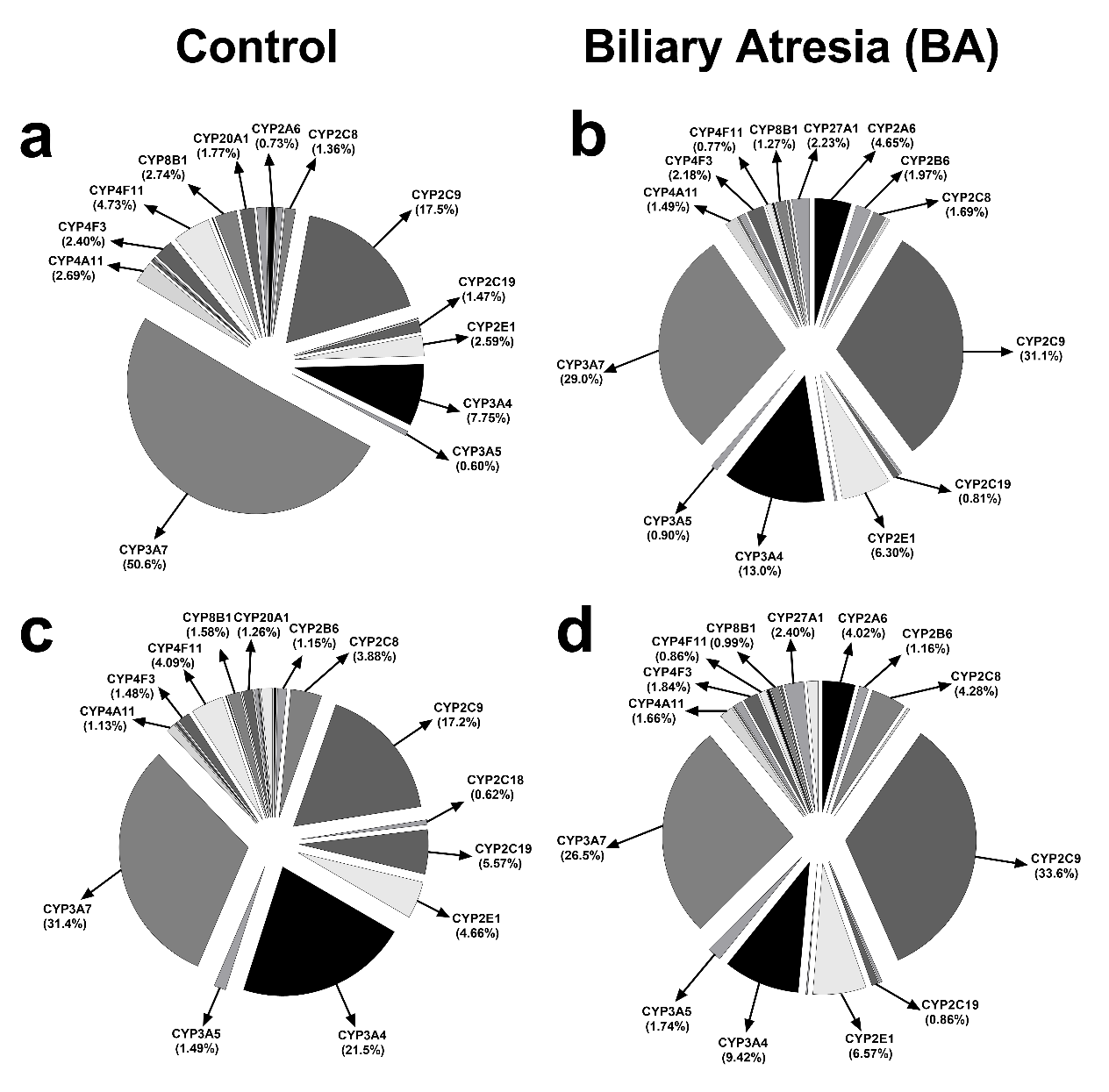
**

**Figure S1**: Distribution of CYP enzyme isoforms in neonatal and infant livers under control and BA conditions. Data are shown for (a) neonatal control livers (n = 13), (b) neonatal BA livers (n = 13), (c) infant control livers (n = 6) and (d) infant BA livers (n = 12). Each segment of the pie chart represents the percentage contribution of individual CYP isoform, highlighting differences in enzyme expression between control and BA groups as well as between neonatal and infant stages.

**Distribution of UGT enzyme in neonatal and infant livers under control and BA conditions**

Pie charts in Figure S2 illustrate the distribution of UGT isoforms in neonatal (a, b) and infant livers (c, d) under control (a, c) and BA (b, d) conditions. In control neonates (Figure S2a), UGT2B15 accounted for 46.9% of expressed UGTs, followed by UGT2B4 (32.6%), and UGT1A1 (12%), with the remaining UGTs contributing less than 10% each. In control infants (Figure S2c), the proportion of UGT2B15 slightly decreased to 39% and UGT2B4 to 26%. Low abundance proteins began to increase, such as UGT1A1, from 12% to 20.6%. The BA groups, and particularly the BA neonate group (Figure S2b), presented similar proportional values to the control neonate group. UGT2B15, UGT2B4 and UGT1A1 remained highly expressed in both groups, and only incremental changes were seen for the remaining proteins. In neonatal control livers (Figure S2a), UGT2B15 was the most dominant isoform. This dominance remains substantial in neonatal and infant BA livers (Figure S2b, 2d), although its proportion slightly decreases to 30.7% and 20%, respectively, likely reflecting shifts in enzyme expression due to the disease state. This reduction in UGT2B15's proportion in BA livers (Figure S2b, d) is accompanied by an increased representation of polymorphic UGT2B15^D85Y^, which is expressed significantly in both BA groups. The reduction in UGT2B15's proportion in BA livers is also accompanied by increased representation of other enzymes, such as UGT2B7, which rose significantly in the BA groups.


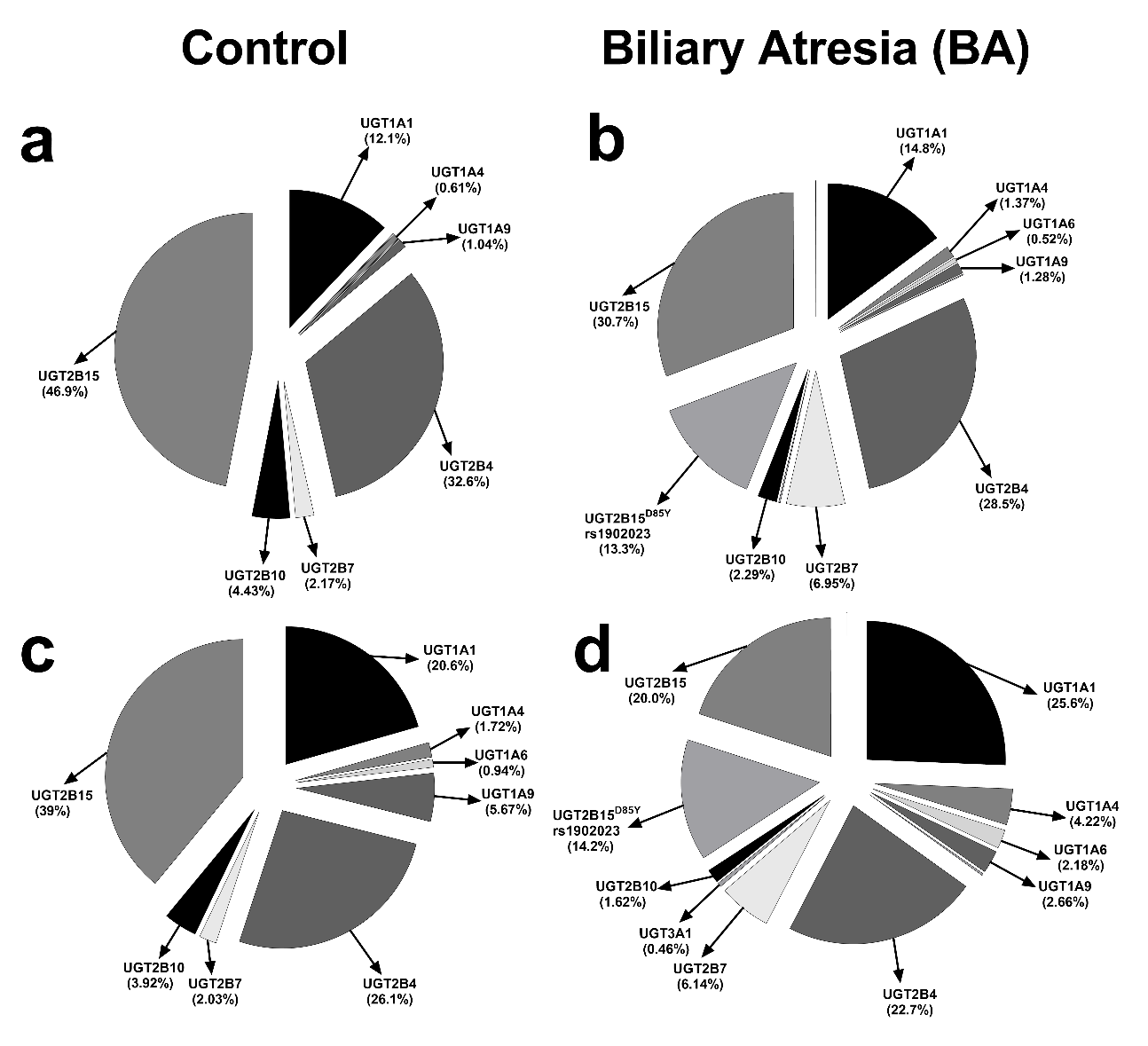


**Figure S2**. Pie charts representing the proportional distribution (%) of various UDP-glucuronosyltransferase (UGT) isoforms in four distinct sample sets: control neonates (a), BA neonates (b), control infants (c), and BA infants (d). Each section of the pie chart corresponds to a specific UGT isoform, with the percentage value indicating its relative abundance within each sample set.

**Proportion of ABC transporters in control and BA livers from neonates and infants**

The pie charts (Figure S3) illustrate the distribution of 10 ABC transporters in control and BA livers from neonates and infants, highlighting transporters with increased or decreased expression levels. In neonatal control livers (Figure S3a), PMF70 (ABCD3) (40%) and ABCA3 (22%) are the most prominent transporters, with ABCE1 contributing moderately at 15.9%. In neonatal BA livers (Figure S3b), a notable reduction in PMF70 expression is observed, decreasing to 21.4%, while ABCF1 became dominant, increasing significantly to 64.7%. This shift suggests non-uniformity of changes to transporter expression in the disease, leading to a different proportional abundance. Other transporters, such as ABCA3, show no expression compared with the control neonatal group (22.1%), reflecting the impact of BA on the neonatal liver's (Figure S3b) transporter profile. In infant livers, similar trends are observed. In the control group (Figure S3c), PMF70 (ABCD3) remains a major transporter at 26.6%, while ABCE1 accounts for 15.4%, and ABCA3 increases to 36%. In BA infant livers (Figure S3d), ABCF1 becomes overwhelmingly dominant at 70.2% (5.4% in control infants), while PMF70 decreases further to 18.2%. ABCE1 shows a decline in BA infant livers compared with the control group from 15.4% to 2.4%, indicating its diminished role in the diseased state. A comparison between neonatal and infant control livers (Figure S3a, S3c) reveals distinct developmental changes in transporter expression. In neonatal control livers, PMF70 and ABCA3 dominate the transporter profile, with PMF70 being the most prominent. In infant control livers, ABCA3 becomes the primary transporter, while PMF70 shows a notable decline. In neonatal BA livers (Figure S3b), ABCF1 dominates, accounting for 64.7% of the total transporters, while PMF70 decreases significantly to 21.4%. In infant BA livers (Figure S3d), ABCF1 remains the most prominent transporter, further increasing to 70.2%, while PMF70 slightly declines to 18.2%. These shifts highlight a consistent pattern of increased ABCF1 expression and reduced PMF70 abundance in BA, leading significant changes to proportional abundance of transporters in the disease state. MRP6 maintained the same relative contribution across disease conditions and developmental stages.


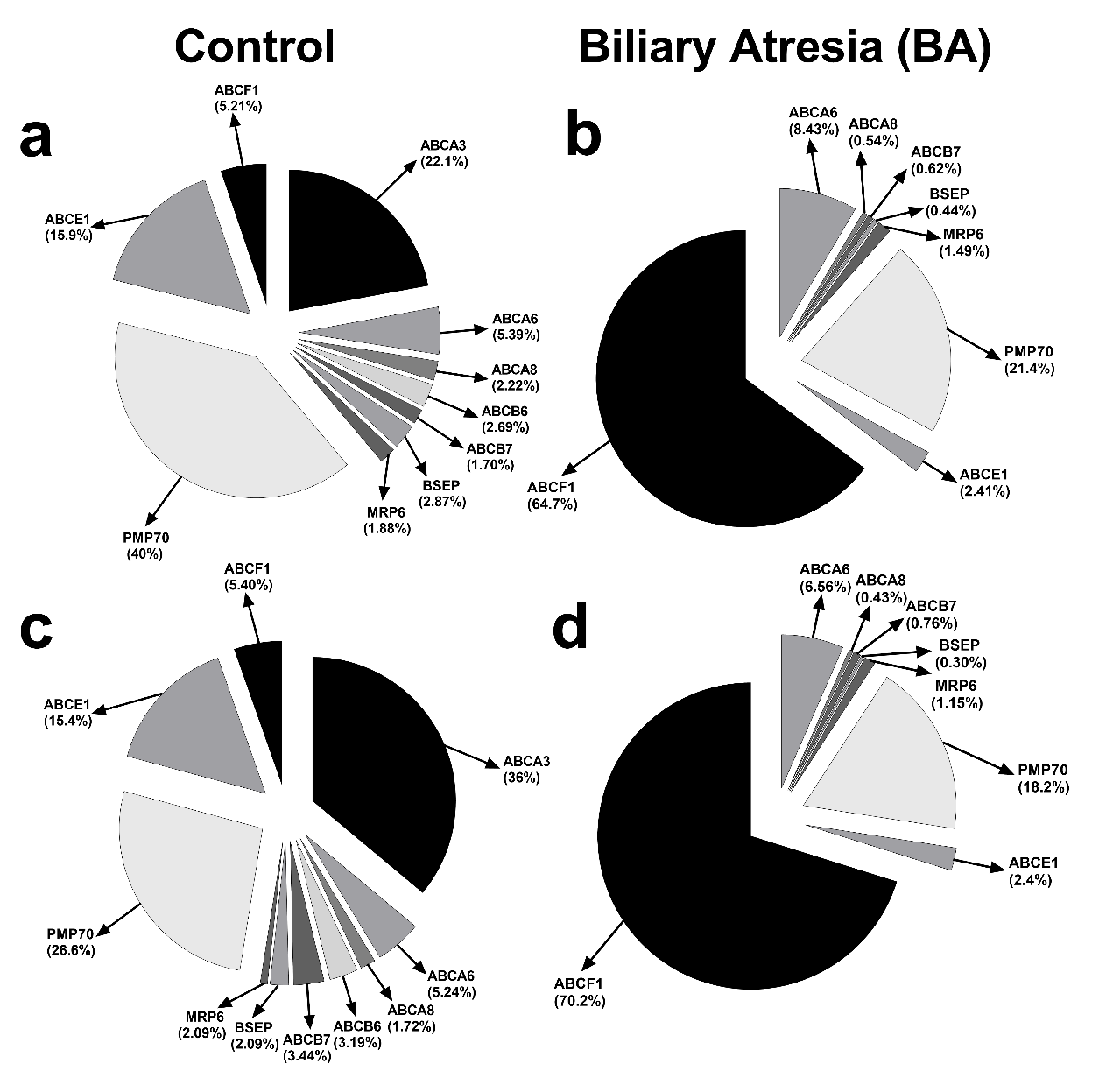


**Figure S3.** Pie charts illustrating the proportional distribution (%) of ABC transporters across four sample groups: control neonates, n=13 (a), BA neonates, n=13 (b), control infants, n=6 (c), and BA infants, n=12 (d). Each segment of the pie chart represents a specific ABC transporter, with the percentage reflecting its relative contribution within the respective sample set.

**Proportion of solute carriers (SLCs) in control and BA livers from neonates and infants**

The pie charts (Figure S4) depict the distribution of SLC transporters in neonatal and infant livers under control and BA conditions. In neonatal control livers (a), SLC27A5 (23.2%) and SLC25A5 (21.9%) are the primary contributors. In neonatal BA livers (b), SLC25A5 increases to 35.7% and becomes the most dominant transporter. In infant control livers (c), the distribution shows a balanced presence of SLC25A15 and SLC25A13, with an increase in SLC25A5 to 34.7%. In infant BA livers (d), SLC25A5 slightly increases to 35.7%, maintaining its dominance, while other transporters show modest adjustments. This shift highlights the significant impact of biliary atresia and developmental stage on the relative expression of SLC transporters, particularly the
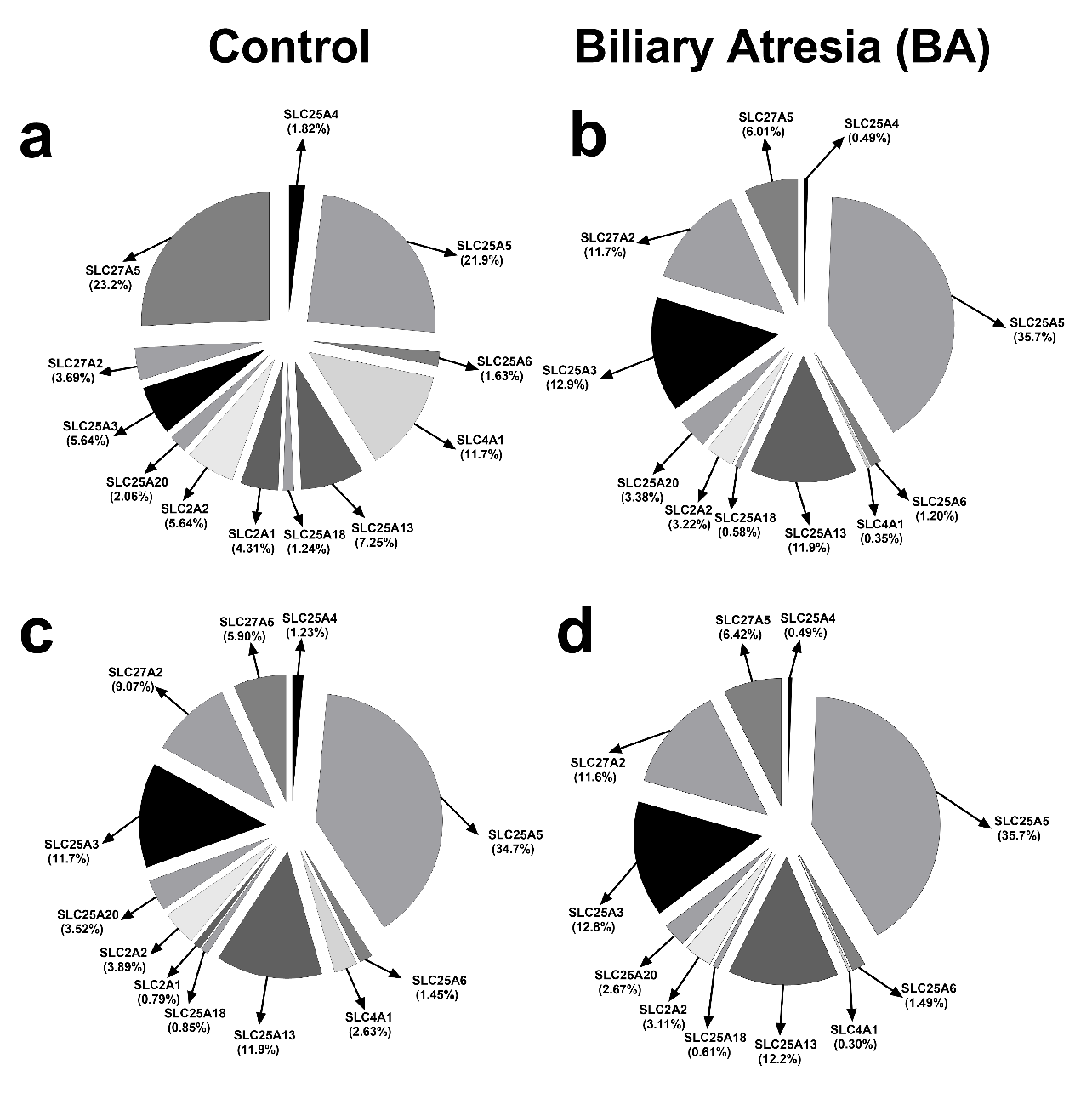
increased prominence of SLC25A5 in diseased livers.

**Figure S4.** Pie charts showing the proportional distribution (%) of SLC transporters in four sample groups: (a) control neonates (n=13), (b) BA neonates (n=13), (c) control infants (n=6), and (d) BA infants (n=12). Each section of the pie charts corresponds to a specific SLC transporter, with the percentage indicating its relative abundance within the respective group.

**Table S3**: Abundance of UDP-glucuronosyltransferase (UGT) enzymes in healthy and BA livers from neonates and infants. Data include the median, mean ± standard deviation (SD), % coefficient of variation (CV), and range of abundance.

| **Enzyme** | **Donors** | **Median (pmol/mg)** | **Mean ± SD (pmol/mg)** | **CV**  **(%)** | **Range (pmol/mg)** |
| --- | --- | --- | --- | --- | --- |
| UGT1A1 | Control Neonate | 1.43 | 1.90 ± 1.73 | 90.8 | 0.49 - 7.20 |
|  | BA Neonate*** | 6.75 | 11.7 ± 10.2 | 86.9 | 1.58 - 31.6 |
|  | Control Infant | 5.25 | 7.64 ± 6.77 | 88.6 | 1.80 - 20.7 |
|  | BA Infant** | 22.1 | 24.7 ± 15.6 | 63.2 | 5.08 - 57.1 |
| UGT1A4 | Control Neonate | 0.07 | 0.10 ± 0.07 | 77.5 | 0.03 - 0.23 |
|  | BA Neonate**** | 0.56 | 1.09 ± 1.22 | 112 | 0.05 - 1.06 |
|  | Control Infant | 0.50 | 0.64 ± 0.63 | 97.7 | 0.07 - 1.78 |
|  | BA Infant** | 2.94 | 4.07 ± 3.59 | 88.3 | 0.53 - 10.9 |
| UGT1A6 | Control Neonate | 0 | 0.01 ± 0.03 | 185.4 | 0.00 - 0.08 |
|  | BA Neonate | 0.41 | 0.42 ± 0.28 | 68.2 | 0.05 - 1.06 |
|  | Control Infant | 0.27 | 0.35 ± 0.33 | 94.8 | 0.02 - 0.85 |
|  | BA Infant** | 1.93 | 2.11 ± 1.51 | 71.6 | 0.24 - 5.10 |
| UGT1A9 | Control Neonate | 0.08 | 0.16 ± 0.29 | 176.6 | 0.01 - 1.10 |
|  | BA Neonate*** | 1.06 | 1.02 ± 0.82 | 80.7 | 0.06 - 2.45 |
|  | Control Infant | 1.65 | 2.11 ± 1.63 | 77.2 | 0.21 - 4.94 |
|  | BA Infant | 2.37 | 2.57 ± 1.81 | 70.5 | 0.34 - 5.59 |
| UGT2A3 | Control Neonate | 0 | 0 | 0 | 0 |
|  | BA Neonate | 0.65 | 0.64 ± 0.31 | 48.9 | 0.05 - 1.35 |
|  | Control Infant | 0 | 0 | 0 | 0 |
|  | BA Infant | 0.60 | 0.89 ± 0.73 | 82.1 | 0.32 - 2.49 |
| UGT2B4 | Control Neonate | 4.04 | 5.13 ± 3.70 | 72.0 | 1.67 - 15.2 |
|  | BA Neonate*** | 21.6 | 22.6 ± 12.1 | 53.7 | 0.56 - 50.0 |
|  | Control Infant | 6.31 | 9.70 ± 7.62 | 78.5 | 5.13 - 24.7 |
|  | BA Infant* | 19.4 | 21.9 ± 12.1 | 55.2 | 8.82 - 41.8 |
| UGT2B7 | Control Neonate | 0.20 | 0.34 ± 0.27 | 77.9 | 0.10 - 0.92 |
|  | BA Neonate**** | 5.32 | 5.50 ± 3.46 | 63.0 | 0.12 - 11.7 |
|  | Control Infant | 0.55 | 0.75 ± 0.73 | 97.4 | 0.23 - 2.21 |
|  | BA Infant*** | 5.12 | 5.92 ± 4.07 | 68.7 | 2.09 - 15.4 |
| UGT2B10 | Control Neonate | 0.71 | 0.70 ± 0.38 | 54.1 | 0.32 - 1.73 |
|  | BA Neonate** | 1.80 | 1.81 ± 0.97 | 53.7 | 0.43 - 3.53 |
|  | Control Infant | 1.22 | 1.46 ± 0.81 | 55.8 | 0.66 - 2.53 |
|  | BA Infant | 1.68 | 1.56 ± 0.66 | 42.4 | 0.40 - 2.67 |
| UGT2B15 | Control Neonate | 4.41 | 7.40 ± 6.41 | 86.6 | 1.94 - 24.7 |
|  | BA Neonate** | 17.7 | 24.3 ± 19.3 | 79.4 | 0.66 - 75.3 |
|  | Control Infant | 10.7 | 14.5 ± 11.8 | 81.2 | 6.12 - 38.1 |
|  | BA Infant | 19.8 | 19.3 ± 12.5 | 64.7 | 3.66 - 47.4 |

Statistical significance is indicated by asterisks; *P < 0.05; **P < 0.01; ***P < 0.001; ****P < 0.0001 vs. corresponding control group (Mann-Whitney U test)

**Table S4**: Abundance of SLC transporters in healthy and BA livers from neonates and infants. Data include the median, mean ± standard deviation (SD), % coefficient of variation (CV), and range of abundance.

| **Protein** | **Donors** | **Median (pmol/mg)** | **Mean ± SD (pmol/mg)** | **CV**  **(%)** | **Range (pmol/mg)** |
| --- | --- | --- | --- | --- | --- |
| SLC2A1 | Control Neonate | 5.25 | 6.45 ± 3.40 | 53 | 2.44 - 12.5 |
|  | BA Neonate | 0 | 0 | 0 | 0 |
|  | Control Infant | 5.08 | 4.73 ± 1.72 | 36 | 2.30 - 6.63 |
|  | BA Infant | 0 | 0 | 0 | 0 |
| SLC2A2 | Control Neonate | 7.11 | 8.44 ± 4.53 | 54 | 1.77 - 16.2 |
|  | BA Neonate* | 4.14 | 4.71 ± 1.98 | 42 | 0.57 - 8.06 |
|  | Control Infant | 9.53 | 9.93 ± 5.42 | 55 | 3.27 - 16.2 |
|  | BA Infant* | 3.39 | 4.74 ± 3.85 | 81 | 2.17 - 15.9 |
| SLC4A1 | Control Neonate | 21.9 | 17.6 ± 8.60 | 49 | 6.03 - 28.2 |
|  | BA Neonate**** | 0.42 | 0.51 ± 0.30 | 58 | 0.03 - 1.04 |
|  | Control Infant | 13.4 | 14.2 ± 7.65 | 54 | 5.03 - 26.7 |
|  | BA Infant*** | 0.51 | 0.47 ± 0.27 | 58 | 0.10 - 0.88 |
| SLC25A3 | Control Neonate | 7.54 | 8.43 ± 5.43 | 64 | 1.52 - 20.3 |
|  | BA Neonate** | 17.1 | 18.8 ± 8.55 | 45 | 0.56 - 35.1 |
|  | Control Infant | 11.2 | 12.5 ± 5.80 | 46 | 5.26 - 19.7 |
|  | BA Infant | 17.6 | 19.5 ± 7.47 | 38 | 8.96 - 32.6 |
| SLC25A4 | Control Neonate | 2.44 | 2.72 ± 1.49 | 55 | 0.74 - 6.01 |
|  | BA Neonate**** | 0.61 | 0.72 ± 0.34 | 47 | 0.04 - 1.24 |
|  | Control Infant | 3.89 | 5.27 ± 3.89 | 74 | 1.51 - 12.3 |
|  | BA Infant*** | 0.63 | 0.75 ± 0.51 | 67 | 0.08 - 1.53 |
| SLC25A5 | Control Neonate | 31.3 | 32.8 ± 14.9 | 45 | 8.93 - 54.8 |
|  | BA Neonate* | 50.8 | 52.1 ± 23.5 | 45 | 1.48 - 96.8 |
|  | Control Infant | 45.4 | 51.5 ± 20.5 | 40 | 30.0 - 82.4 |
|  | BA Infant | 57.3 | 54.4 ± 18.2 | 33 | 25.8 - 77.6 |
| SLC25A13 | Control Neonate | 9.09 | 10.8 ± 6.85 | 63 | 1.31 - 23.9 |
|  | BA Neonate* | 14.6 | 17.5 ± 7.55 | 43 | 3.09 - 29.1 |
|  | Control Infant | 18.9 | 18.8 ± 8.09 | 43 | 4.27 - 27.6 |
|  | BA Infant | 17.1 | 18.5 ± 7.42 | 40 | 8.74 - 30.6 |
| SLC27A2 | Control Neonate | 5.53 | 5.52 ± 3.46 | 63 | 1.45 - 12.5 |
|  | BA Neonate**** | 16.0 | 17.1 ± 6.53 | 38 | 2.41 - 27.7 |
|  | Control Infant | 3.85 | 4.26 ± 1.61 | 38 | 2.16 - 6.92 |
|  | BA Infant*** | 18.0 | 17.8 ± 6.0 | 34 | 7.40 - 29.2 |
| SLC27A5 | Control Neonate | 23.1 | 34.7 ± 27.3 | 79 | 14.4 - 115 |
|  | BA Neonate**** | 7.36 | 8.78 ± 4.97 | 57 | 4.06 - 23.1 |
|  | Control Infant | 9.33 | 9.92 ± 4.98 | 50 | 2.99 - 16.4 |
|  | BA Infant | 9.32 | 9.79 ± 4.50 | 46 | 4.16 - 17.8 |
| SLCO1B1 | Control Neonate | 0.49 | 0.47 ± 0.24 | 50 | 0.04 - 0.89 |
|  | BA Neonate*** | 0.14 | 0.14 ± 0.10 | 69 | 0.00 - 0.31 |
|  | Control Infant | 0.48 | 0.56 ± 0.29 | 52 | 0.29 - 1.12 |
|  | BA Infant*** | 0.11 | 0.14 ± 0.10 | 70 | 0.07 - 0.44 |

Statistical significance is indicated by asterisks; *P < 0.05; **P < 0.01; ***P < 0.001; ****P < 0.0001 vs. corresponding control group (Mann–Whitney U test)

**References**

1. Vasilogianni AM, Al-Majdoub ZM, Achour B, et al. Quantitative Proteomics of Hepatic Drug-Metabolizing Enzymes and Transporters in Patients With Colorectal Cancer Metastasis. Clin Pharmacol Ther 2022;112(3):699-710.

2. Achour B, Al Feteisi H, Lanucara F, Rostami-Hodjegan A, Barber J. Global Proteomic Analysis of Human Liver Microsomes: Rapid Characterization and Quantification of Hepatic Drug-Metabolizing Enzymes. Drug Metab Dispos 2017;45(6):666-75.

3. Howard M, Achour B, Al-Majdoub Z, Rostami-Hodjegan A, Barber J. GASP and FASP are Complementary for LC-MS/MS Proteomic Analysis of Drug-Metabolizing Enzymes and Transporters in Pig Liver. Proteomics 2018;18(24):e1800200.

4. Al-Majdoub ZM, Scotcher D, Achour B, et al. Quantitative Proteomic Map of Enzymes and Transporters in the Human Kidney: Stepping Closer to Mechanistic Kidney Models to Define Local Kinetics. Clin Pharmacol Ther 2021;110(5):1389-400.

5. Al-Majdoub ZM, Carroll KM, Gaskell SJ, Barber J. Quantification of the proteins of the bacterial ribosome using QconCAT technology. J Proteome Res 2014;13(3):1211-22.

6. Al-Majdoub ZM, Al Feteisi H, Achour B, et al. Proteomic Quantification of Human Blood-Brain Barrier SLC and ABC Transporters in Healthy Individuals and Dementia Patients. Mol Pharm 2019;16(3):1220-33.

7. Al-Majdoub ZM, Achour B, Couto N, et al. Mass spectrometry-based abundance atlas of ABC transporters in human liver, gut, kidney, brain and skin. FEBS Lett 2020;594(23):4134-50.
